# Total Synthesis, Stereochemical Assignment, and Divergent Enantioselective Enzymatic Recognition of Larreatricin

**DOI:** 10.1002/chem.201803785

**Published:** 2018-10-01

**Authors:** Harry J. Martin, Ioannis Kampatsikas, Rik Oost, Matthias Pretzler, Emir Al‐Sayed, Alexander Roller, Gerald Giester, Annette Rompel, Nuno Maulide

**Affiliations:** ^1^ Institut für Organische Chemie Universität Wien Währinger Straße 38 1090 Wien Austria; ^2^ Institut für Biophysikalische Chemie Universität Wien Althanstraße 14 1090 Wien Austria; ^3^ Zentrum für Röntgenstrukturanalyse Universität Wien Währinger Straße 42 1090 Wien Austria; ^4^ Institut für Mineralogie und Kristallographie Universität Wien Althanstraße 14 1090 Wien Austria

**Keywords:** enantiospecific, kinetics, larreatricin, polyphenol oxidase, total synthesis

## Abstract

A concise and efficient total synthesis of the lignan natural product larreatricin as well as an unambiguous assignment of configuration of its enantiomers are reported, resolving a long‐held controversy. Enzyme kinetic studies revealed that different polyphenol oxidases show high and remarkably divergent enantioselective recognition of this secondary metabolite.

Chirality is a ubiquitous feature of biological systems that plays a critical role in the metabolic processes of living organisms.^1^ In particular, enzymatic reactions are highly stereospecific.^2^ Polyphenol oxidases (PPOs) are enzymes ubiquitous across a wide range of organisms, from bacteria to fungi, plants and also mammals,^3^, ^4^, ^5^ that contain a type‐III copper center in their active site.^6^ Most PPOs are expressed as latent proenzymes in vivo containing a catalytically active domain and a C‐terminal domain.^7^ This C‐terminal domain shields the active site of the PPO and, as a result, the enzymes either possess only very weak or even no enzymatic activity unless they are activated by, for example, proteases, an acidic pH, fatty acids, or detergents (e.g., sodium dodecyl sulfate, SDS).^8^, ^9^, ^10^ The precise physiological roles of PPOs have hitherto been largely difficult to elucidate, especially because they accept a variety of aromatic substrates. This has led to considerable uncertainty about the roles played by these enzymes in vivo.^6^ Tyrosinases (TYRs, EC 1.14.18.1 and EC 1.10.3.1), a subgroup of the PPO family, use molecular oxygen to catalyze the *ortho*‐hydroxylation of monophenols to catechols coupled with the subsequent oxidation of catechols to *o*‐quinones.^4^, ^5^ The latter quinones are highly reactive and thus lead to the non‐enzymatic formation of insoluble pigments like melanin. The best characterized PPOs are those from mushrooms, among which *Ab*PPO4^11^ is reported to preferentially convert l‐tyrosine over d‐tyrosine.11a The enantiospecificity of PPOs or how PPOs select one enantiomer of a substrate over the other remain poorly investigated. They constitute open topics of high relevance for the understanding of how PPOs function in vivo and also how their enzymatic specificity may be tailored to provide an efficient tool for the enantioselective *o*‐hydroxylation of a very broad range of phenolic compounds.

The naturally occurring lignan larreatricin **1** (Scheme 1 A) belongs to a family of structurally related compounds occurring in the creosote bush *Larrea tridentata*.^12^ This plant has a history of use in traditional South American medicine for various ailments including rheumatism, venereal diseases and digestive disorders. Other secondary metabolites derived thereof include the powerful antioxidant nordihydroguaiaretic acid (NDGA) depicted in Scheme 1 A. NDGA has potential applications in the treatment of a number of diseases, including various types of cancer, cardiovascular diseases and neurological disorders and it is widely used for the treatment of actinic keratoses.^12^ In 2003, Lewis reported that the enzyme (+)‐larreatricin hydroxylase, isolated from *Larrea tridentata*, selectively accepts only the (+)‐enantiomer of larreatricin as a substrate.^12^

**Scheme 1:**
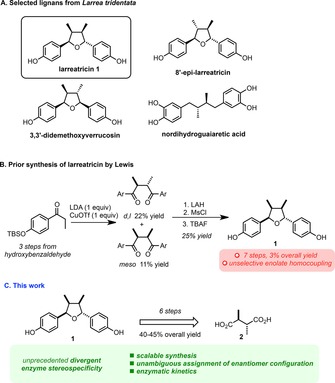
(A) Lignan metabolites from *Larrea tridentate*, (B) prior low‐yielding synthesis of **1** and (C) proposed scalable synthesis and enzymatic studies.

In the same year, the same authors reported a racemic synthesis of larreatricin by using a low‐yielding oxidative enolate homocoupling as the key‐step (Scheme 1 B). Unfortunately, the overall poor yield of 3 % over seven steps^13^ and the absence of stereoselectivity (a mixture of all possible isomers was produced, with no apparent selectivity for the title compound), significantly hindered further investigation of this intriguing family of natural products. Additionally, an unambiguous assignment of the absolute configuration of naturally occurring (−)‐larreatricin is still missing after nearly three decades of research on this family of metabolites.^14^

Given our prior interest in the stereoselective synthesis of tetrahydrofuran derivatives^15^ and in the chemistry and biology of PPOs^6^ from a variety of plants,^16^, ^17^, ^18^ our collaborative interest was piqued by the enantiospecificity of larreatricin hydroxylase and the possibility that other tyrosinases might exhibit similar behavior. Here, we report a concise, efficient and scalable enantioselective synthesis of **1** and an unambiguous structural assignment of its (+) and (−) enantiomeric forms. We also report detailed kinetic studies of its divergent enantiospecific uptake by three different tyrosinases from two kingdoms (Scheme 1 C„ Table 1), including (+)‐larreatricin hydroxylase, which we were able to produce recombinantly for the first time.

Larreatricin features some interesting symmetry elements. Of particular relevance for our retrosynthetic analysis are the vicinal methyl groups, which form a chirotopic non‐stereogenic unit. Interestingly, the overall 2,3‐diaryl‐3,4‐dimethyl‐tetrahydrofuran core itself allows for two possible *meso* configurations and four chiral arrangements (two each of *C*_1_ and *C*_2_ symmetry). These considerations inspired us to begin our synthesis from a *meso* precursor, that is, the *meso* isomer of 2,3‐dimethylsuccinic acid **2** (Scheme 2).

**Scheme 2:**
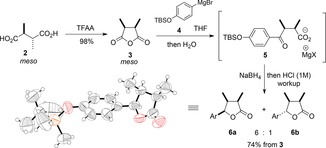
Synthesis of stereodefined trisubstituted lactone **6 a**; TFAA=trifluoroacetic anhydride; Ar=*p*‐TBSO‐C_6_H_4,_ TBSO=*tert*‐butyldimethylsilyl ether.

This commercially available diacid was cyclodehydrated to the *meso* anhydride **3** 19 in a nearly quantitative yield by using trifluoroacetic anhydride (Scheme 2, top). The anhydride was then subjected to nucleophilic ring‐opening by the Grignard reagent **4**, derived from silyl‐protected 4‐bromophenol. We quickly ascertained the lability of the ketoacid that is generated in this transformation because all the attempts to isolate it led to a significant loss of material even though its formation appeared to proceed with high chemical yield. It was thus deemed advantageous to carry out the reduction of the magnesium carboxylate **5** in situ by using sodium borohydride followed by acidic (HCl) workup. This simple and scalable sequence led to a 6:1 diastereomeric mixture of trisubstituted lactones, of which all‐*syn* **6 a** was the major component (Scheme 2). The isomer **6 a** is a crystalline material and its structure and relative stereochemical assignment were confirmed by X‐ray crystallographic analysis (CCDC https://summary.ccdc.cam.ac.uk/structure-summary?doi=10.1002/chem.201803785 contains the supplementary crystallographic data for this paper. These data are provided free of charge by http://www.ccdc.cam.ac.uk/). Importantly, the overall sequence from diacid **2** to trisubstituted lactone **6 a** required a single chromatographic purification, delivering the pure material in 62 % overall yield over the three steps on a multi‐gram scale.

At this stage, we envisaged a lactone partial reduction—easily achieved inquantitative yield using diisobutylaluminium hydride (DIBAL‐H) Scheme 3—followed by substitution of the lactol **7**/acetal **8** with the second aryl nucleophilic equivalent. However, several attempts employing a range of Lewis acids and organometallic nucleophiles delivered not only **1** (<5 %), but also multiple isomeric natural product congeners (such as those shown in Scheme 1 A) in an overall modest yield. If we assume that the reaction proceeds via the oxacarbenium ion **9**, the preferred trajectory for attack of the incoming nucleophile should be *anti* to the C‐3 methyl group for both conformers and thus deliver the desired configuration. The formation of multiple isomers instead suggests that either (a) the oxacarbenium **9** quickly epimerizes (via the enol ether) competitively with nucleophilic addition, or (b) the tetrahydrofuran **10** undergoes further loss of configurational purity and/or destruction initiated by the formation of the *p*‐quinone methide through Lewis acid‐mediated ring‐opening.^20^

**Scheme 3:**
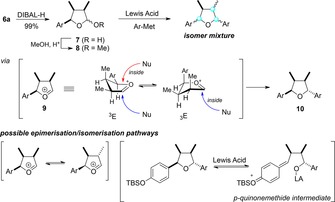
Unsuccessful attempts at installing the fourth substituent; DiBAL‐H=diisobutylaluminium hydride.

The need to prevent epimerization and side reactions suggested structurally modifying the leaving group at carbon C‐2, to avoid the need for a strong Lewis acid. After some experimentation (Scheme 4), we found that the conversion of lactol **7** to a sulfone acetal **11**,^21^ followed by the addition of the Grignard reagent **4** in the presence of zinc(II) bromide gratifyingly generated the tetrasubstituted tetrahydrofuran **10** 13 as the main diastereomeric species in 70–80 % yield from lactol **7**.^22^ The desilylation finally afforded *rac*‐**1** in six steps and an overall yield of 37–45 % from the commercially available succinic acid **2**. This is a significant improvement in efficiency and practicality when compared to the prior reports.^13^ Chiral HPLC separation provided samples of both (−)‐ and (+)‐larreatricin. It was important to obtain and characterize both enantiomers for subsequent structural and enzymatic studies (vide infra).

**Scheme 4:**
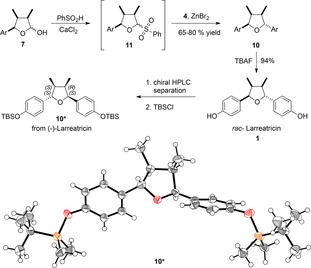
Completion of the synthesis of **1** and assignment of the absolute configuration of (−)‐**1** by X‐ray crystallography; Ar=*p*‐TBSO‐C_6_H_5_, TBSO=*tert*‐butyldimethylsilyl ether; TBAF=tetrabutylammonium fluoride.

Notably, the separation of enantiomers also enabled us to unambiguously establish the absolute configuration of (−)‐larreatricin by X‐ray crystallographic analysis of the silylated derivative 10* (CCDC https://summary.ccdc.cam.ac.uk/structure-summary?doi=10.1002/chem.201803785 contains the supplementary crystallographic data for this paper. These data are provided free of charge by http://www.ccdc.cam.ac.uk/). As shown in Scheme 4, (−)‐larreatricin has a (2*S*, 5*S*)‐configuration. This is, to the best of our knowledge, the first unambiguous stereochemical assignment of this natural product.

In parallel, we developed an enantioselective synthesis of the key lactone (+)‐**6 a** (Scheme 5) by using an anhydride desymmetrization approach pioneered by Rovis.^23^ In situ reduction of the carboxylate (as before) was not possible following this protocol, and the aforementioned instability of (+)‐**12** is responsible for its modest isolated yield. Notably, the ketone reduction in this sequence is also very sensitive to reaction conditions giving access to both diastereomers.^24^ Nonetheless, the reduction of (+)‐**12** with DIBAL‐H afforded (+)‐**6 a** in high enantioselectivity and diastereoselectivity comparable to the racemic pathway. Thus, this establishes an enantioselective route for the total synthesis of (+)‐larreatricin.

**Scheme 5:**
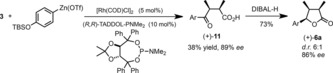
Enantioselective desymmetrization of **3** en route to (+)‐**1**.

At this juncture, we investigated the enantioselectivity towards the substrate larreatricin for three recombinantly expressed PPOs. We employed 1) the only described enantiospecific plant enzyme larreatricin hydroxylase from *Larrea tridentata* (*Lt*PPO),^12^ for which we established a heterologous production protocol yielding approximately 20 mg of protein per liter of bacterial culture; 2) the apple tyrosinase (*Md*PPO1);16b and 3) the fungal PPO from *Agaricus bisporus* (*Ab*PPO4),11a for which also the proteolytically activated form (*Ab*PPO4‐act)11a was investigated (Supporting Information, Figure S4). This activated form is derived from *Ab*PPO4 by removal of the C‐terminal domain, which relieves the latency of the PPO.11a Therefore, this allows for the direct comparison of the enzymatic activity liberated by treatment with a detergent to the enzymatic reaction carried out in the absence of any external activator.

The enzymatic *o*‐hydroxylation and oxidation of phenolic compounds by PPOs gives rise to reactive *o*‐quinones that usually evolve dark pigments of the melanin family.^25^ Considering that these reactions can complicate the kinetic assay, we applied the potent nucleophile 3‐methyl‐2‐benzothiazolinone hydrazone (MBTH) as a chinone‐trapping agent that yields soluble and reasonably stable coupling products.^26^ The three enzymes revealed completely different enantiospecificities for (+)‐ and (−)‐larreatricin (Table 1). The lag‐phase usually encountered when monitoring the PPO reaction on monophenolic substrates^27^ was shorter than two seconds for the preferred enantiomer of larreatricin for each of the four tested enzyme preparations. For the non‐preferred substrates, it took up to one minute until the steady‐state rate of the reaction was reached; the substrate conversion at this point did not exceed 15 % of the initial substrate concentration. *Lt*PPO displayed a clear preference for (+)‐larreatricin and the *k*_cat_ value was 24 times higher than for the reaction with (−)‐larreatricin (*k*_cat_=11.3 s^−1^ and 0.48 s^−1^, respectively). This affirms the previously observed enantiospecificity of the enzyme,^12^ but our results show that (−)‐**1** is also accepted as a substrate by *Lt*PPO. The preference of *Lt*PPO for (+)‐larreatricin over the (−)‐enantiomer clearly indicates that the first is the natural substrate of *Lt*PPO. The *K*_M_ values for (+)‐ and (−)‐**1**, 0.062 mm and 0.27 mm, respectively, and the 62 μm for (+)‐larreatricin are among the lowest *K*_M_ values reported for PPOs, pointing towards a very strong enzyme–substrate interaction.

**Table 1:** Kinetic characterization of the reaction of larreatricin with three PPOs.^[a]^

Enzyme	Larreatricin	*K*_M_ [mm]	*k*_cat_ [s^−1^]
*Lt*PPO^[b]^	(+)	0.062±0.0044	11.3±0.58
	(−)	0.27±0.020	0.48±0.02
*Md*PPO116b	(+)	0.21±0.019	0.61±0.02
	(−)	0.34±0.031	0.34±0.02
*Ab*PPO411a	(+)	0.25±0.036	1.14±0.12
	(−)	0.28±0.025	14.9±0.93
*Ab*PPO4‐act11a	(+)	0.11±0.010	0.91±0.033
	(−)	0.31±0.072	12.1±1.8

[a] The volumetric activity was determined by following the appearance of the quinone adduct, measured photometrically at *λ*_max_=501 nm, *ϵ* (*λ*_max_)=37 mm ^−1^ cm^−1^. [b] This work.

The second plant enzyme *Md*PPO1 showed no clear preference for either one of the enantiomers with the *k*_cat_ on (+)‐larreatricin being 0.61 s^−1^, which is only 1.8 times higher than the *k*_cat_ for the (−)‐enantiomer (*k*_cat_=0.34 s^−1^). The specificity of *Md*PPO1 is also similar for the two enantiomers, with the *K*_M_ value for (−)‐larreatricin (0.34 mm) being a little higher than for (+)‐larreatricin (*K*_M_=0.21 mm).

Surprisingly, the mushroom enzyme *Ab*PPO4 exhibited a reversed specificity for the two enantiomers of larreatricin. It reacted on (−)‐larreatricin (*k*_cat_=15 s^−1^) 13 times faster than on (+)‐larreatricin (*k*_cat_=1.1 s^−1^). The *K*_M_ value was found to be 0.25 mm for (+)‐larreatricin and 0.28 mm for (−)‐larreatricin, which indicates a very similar specificity. In the case of *Ab*PPO4, we compared the findings for the latent enzyme with the active enzyme (termed *Ab*PPO4‐act)11a that showed very similar kinetic parameters and was also 13 times faster on the (+)‐enantiomer (Table 1).

These enantioselectivities are among the highest stereoselectivities reported for PPOs so far. For the most well‐studied system, the tyrosinase isolated from mushroom (*Agaricus bisporus*), no significant preference for either *l*‐ or *d*‐tyrosine was found.^28^ A slight preference for l‐DOPA was displayed by the tyrosinase from the bacterium *Bacillus megaterium* that reacted 1.7 times faster on the *S*‐enantiomer than on *R*‐DOPA.^29^ Another bacterial tyrosinase from *Ralstonia solanacearum* showed a medium enantiopreference for l‐tyrosine (4.7 times faster than on the *R*‐enantiomer) which could be reduced to almost no selectivity (0.98, that is, a very slight preference for the *R*‐enantiomer) by mutation of one amino acid far from the active site.^30^ Enantioselectivities similar to the values presented here for larreatricin were observed for a secreted fungal tyrosinase from *Trichoderma reesei* that reacted 14.3 times faster on 2.5 mm *l*‐tyrosine than on its *R*‐enantiomer (based on specific activities because no kinetic characterization was given).^31^ The highest enantioselectivity reported for a PPO comes from tyrosinase produced by the bacterium *Streptomyces sp*. REN‐21 that displayed resistance to organic solvents and was 35.9 times faster on l‐ than on d‐tyrosine.^32^ The enantioselectivity in the reaction with larreatricin is determined to a large extent by the special geometry of larreatricin, which is a rather large substrate. This is indicated by the comparison of our results with those obtained by using those three bacterial and two fungal tyrosinases and especially by the increase in apparent stereoselectivity of *Ab*PPO4 when going from the simple amino acid tyrosine (2.6‐fold faster on the *S*‐enantiomer) to the lignan larreatricine (13 times faster reaction on the (−)‐enantiomer). For *Lt*PPO, the preferred enantiomer of larreatricin also exhibits the smaller *K*_M_ value as would be expected for tighter binding. Although the difference in *K*_M_ is small to negligible for *Md*PPO1 and *Ab*PPO4, a different trend is noted. The enantiomer that undergoes catalysis faster has the higher (even though still low) *K*_M_ value, which usually indicates less favorable binding to the active site. This hints towards a second factor determining the reaction rate besides the accurate fit of the substrate to the active site of the enzyme. The notable difference in the preference for the (+)‐enantiomer exhibited by the enzymes *Lt*PPO on the one hand, and for the (−)‐enantiomer by *Ab*PPO4 on the other hand, gives a clear indication that the active center of PPOs can discriminate between enantiomeric substrates. The generally low *K*_M_ values point towards a strongly bound substrate.

In conclusion, we have reported a concise and efficient total synthesis of the lignan natural product larreatricin as well as the unambiguous assignment of the absolute configuration of its enantiomers. Our synthesis also enabled detailed enzyme kinetic studies with different classes of PPOs, revealing a high and remarkably divergent enantioselectivity in their recognition of this natural product, providing an intriguing basis for further studies of the enzyme–substrate binding mode in the PPO family.

## Conflict of interest

The authors declare no conflict of interest.

## Supporting information

As a service to our authors and readers, this journal provides supporting information supplied by the authors. Such materials are peer reviewed and may be re‐organized for online delivery, but are not copy‐edited or typeset. Technical support issues arising from supporting information (other than missing files) should be addressed to the authors.

Supplementary
