# Supplementary material for: Total Synthesis, Stereochemical Assignment, and Divergent Enantioselective Enzymatic Recognition of Larreatricin
Source: Chemistry. 2018 Oct 1;24(59):15756–60. doi: 10.1002/chem.201803785 (PMC6220842; doi:10.1002/chem.201803785)
Supplement: Supplementary file 1 — Supplementary [file CHEM-24-15756-s001.pdf]

CHEMISTRY
A European Journal

Supporting Information

Total Synthesis, Stereochemical Assignment, and Divergent Enantioselective Enzymatic Recognition of Larreatricin

Harry J. Martin,*[a] Ioannis Kampatsikas+,[b] Rik Oost+,[a] Matthias Pretzler+,[b] Emir Al-Sayed,[b] Alexander Roller,[c] Gerald Giester,[d] Annette Rompel,*[b] and Nuno Maulide*[a]

chem_201803785_sm_miscellaneous_information.pdf

Abstract: A concise and efficient total synthesis of the lignan natural product larreatricin as well as an unambiguous assignment of configuration of its enantiomers are reported, resolving a long-held controversy. Enzyme kinetic studies revealed that different polyphenol oxidases show high and remarkably divergent enantioselective recognition of this secondary metabolite.

Table of Contents

PART A (Chemical Synthesis of Larreatricin)

1. General Procedures, Materials and Instrumentation S-3
2. Experimental Procedures and Characterization Data for the Racemic Synthesis of Larreatricin S-3
3. Experimental Procedures for the Enantioselective Synthesis of Key Compound **6a** S-6
4. NMR Spectra S-9
5. HPLC Reports S-11
6. X-ray Crystallographic Data S-14

PART B (Polyphenol Oxidase Production and Kinetic Characterization with Larreatricin Enantiomers) S-17

1. Plant Material, Cloning and Sequencing of Larreatricin Hydroxylase. S-17
2. Heterologous Expression and Purification of recombinant Larreatricin Hydroxylase S-17
3. Heterologous Expression and Purification of recombinant PPO1 from *Malus domestica* (*Md*PPO1) S-18
4. Heterologous Expression, Purification and Activation of recombinant PPO4 from *Agaricus bisporus* (*Ab*PPO4)........ S-18
5. Enzyme Kinetics and Enzyme Activity Assays. S-18
6. Tables and Figures. S-19

References........ S-21

PART A (Chemical Synthesis of Larreatricin)

1. General Procedures, Materials and Instrumentation

General procedures. All reactions were performed in round bottom flasks or vials fitted with rubber septa with magnetic stirring, unless otherwise stated. Liquids and solutions were transferred via syringe. All reactions were performed using anhydrous solvents from Sigma-Aldrich. Reaction progress was monitored by thin layer chromatography (TLC) performed on glass plates coated with silica gel F 254 with 0.2 mm thickness. Chromatograms were visualized by fluorescence quenching with UV light at 254 nm or by staining using Molybate solutions followed by heating. Flash column chromatography was carried out on 230-400 mesh silica gel (Merck and Co.) using distilled reagent grade solvents.

Materials. All commercial reagents and solvents were used without further purification.

Instrumentation. Infrared (IR) spectra were obtained using Perkin-Elmer Spectrum 100 FT-IR spectrometer. Wavenumbers (v= 1/λ) are reported in cm^{-1}. ^{1}H NMR and ^{13}C NMR spectra were recorded using a Bruker AV-400, AV-600 or AV-700 spectrometer at 300K. Chemical shifts are given in parts per million (ppm, δ), referenced to the solvent peak defined at δ = 7.26 ppm (^{1}H NMR) and δ = 77.16 (^{13}C NMR) for $CDCl_3$ and δ = 2.05 (^{1}H NMR) and δ =29.84 (^{13}C NMR) for acetone-d_6. Coupling constants (J) are reported in Hertz (Hz). ^{1}H NMR splitting patterns are designated as singlet (s), doublet (d), triplet (t), quadruplet (q) and quintuplet (qt). Splitting patterns that could not be unambiguously interpreted were designated as multiplet (m) or broad (br). Optical rotations were measured on a Perkin Elmer 341 polarimeter using a 100 mm path-length cell at 589 nm (c given in g/100 mL). Infrared (IR) spectra were obtained using a Perkin-Elmer Spectrum 100 FT-IR spectrometer. Wavenumbers (v= 1/λ) are reported in cm^{-1}. Mass spectra were obtained using a Finnigan MAT 8200 or (70 eV) or an Agilent 5973 (70 eV) spectrometer, using electrospray ionization (ESI). Chiral HPLC was performed using AGILENT Infinity 1260 with Chiralpak I, Chiralpak IC, or Lux-3 Cellulose-3 columns. Details of chromatographic conditions are indicated under each compound.

2. Experimental Procedures for the Synthesis of *rac*-Larreatricin

***meso*-3,4-Dimethyldihydrofuran-2,5-dione 3**

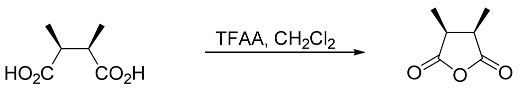

After a literature procedure.[1] A solution of *meso*-2,3-dimethylsuccinic acid (628 mg, 4.3 mmol) was dissolved in 10 mL dry dichloromethane. The solution was cooled to 0 °C and trifluoroacetic anhydride (0.66 mL, 4.73 mmol, 1.1 equiv.) was added dropwise. The reaction was stirred for 2 hours at 0 °C and the solvent was evaporated in *vacuo* to afford the product as a white solid (518 mg, 94%). **^{1}H NMR** (400 MHz, $CDCl_3$) δ 3.22 (m, 2H), 1.31 (m, 6H); **^{13}C NMR** (100 MHz, $CDCl_3$) δ 174.0, 39.0, 11.3. The analytical data are in good accordance with those reported in literature.[2]

(4-Bromophenoxy)(*tert*-butyl)dimethylsilane

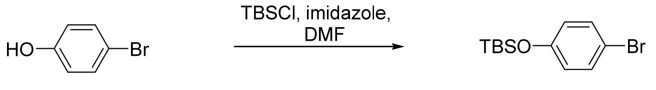

To a solution of 4-bromophenol (10,0 g, 58 mmol) and imidazole (9.86 g, 145 mmol) in DMF (60 mL) was added a solution of TBSCl (11.58 g, 77 mmol) in DMF (40 mL) via dropping funnel under argon atmosphere. The mixture was stirred 48 h and then quenched by adding it to water (400 mL). After extraction with EtOAc (4 x 50 mL) the combined organic layers were washed with brine (100 mL), dried ($MgSO_4$) and the solvents were evaporated at elevated temperature (60 °C) in *vacuo* (1 mbar) until complete removal of the silanol by-product (ca. 3 h) to give the aryl bromide (15.96 g, 96%) as a nearly colorless oil. Further purification by column chromatography (heptane/EtOAc 20:1) was possible but not necessary. **^{1}H NMR** (400 MHz, $CDCl_3$) δ 7.34-7.32 (m, 2H), 7.31-7.29 (m, 2), 0.97 (s, 9H), 0.18 (s, 6H). The analytical data are in good accordance with those reported in literature.[3]

5-(4-((*tert*-Butyldimethylsilyl)oxy)phenyl)-3,4-dimethyldihydrofuran-2(3*H*)-one 6a

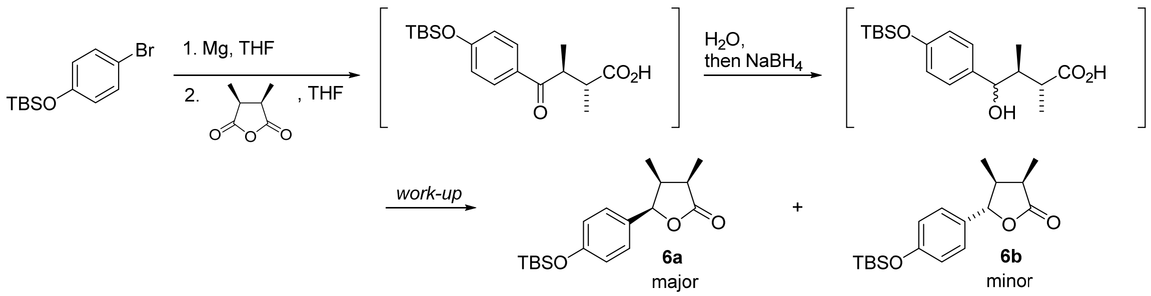

To a solution of the aryl bromide (3.160 g, 11.0 mmol) in dry THF (12 mL) under argon atmosphere, magnesium turnings (0.265 g, 10.5 mmol) were added along with an initiating grain of iodine. After the reaction had started the solution became greyish and was refluxed for 2.5 h before 8 mL of dry THF were added for a better solubility while the whole solution was cooled to 15 °C. The Grignard solution was slowly added to a solution of anhydride **3** (1.280 g, 10.0 mmol) in dry THF (10 mL) at 0 °C. The solution was stirred for 20 min at 0 °C and then for 30 min at 45 °C. After being cooled to 0 °C, 10 mL water were added to give a clear yellow solution. Sodium borohydride (760 mg, 20.08 mmol) was added in small portions over a period of 30 min at 0 °C. The solution was stirred for further 30 min at 0 °C and then at room temperature for 30 min. The reaction mixture was quenched with 250 mL of HCl (1 M) and then extracted with diethyl ether (3 x 100 mL). The organic phases were combined, dried ($MgSO_4$) and slowly evaporated to dryness in vacuum at 40 °C. The obtained residue was reconstituted in a minimum amount of heptane/EtOAc, 5:1, and purified by silica column chromatography (Heptane/EtOAc, 7:1-5:1) to afford **6a** as a solid (2.038 g, 6.36 mmol) along with **6b** (0.332 g, 1.04 mmol) as a colorless oil in an overall yield of 74%. The major isomer **6a** was recrystallized from heptane (m.p. = 99-100 °C) to remove traces of the minor isomer. **6a (**main isomer): **^{1}H NMR** (600 MHz, $CDCl_3$) δ 7.13 (d, J = 8.4 Hz, 2H), 6.84 (d, J = 8.4 Hz, 2H), 5.47 (d, J = 5.2 Hz, 1H), 2.99 (qt, J = 7.2 Hz, 1H), 2.72 (ddq, J = 5.2, 7.2, 7.2 Hz, 1H), 1.21 (d, J = 7.2 Hz, 3H), 0.98 (s, 9H), 0.54 (d, J = 7.2 Hz, 3H), 0.19 (s, 6H). **^{13}C NMR** (150 MHz, $CDCl_3$) δ 179.3, 155.4, 128.9, 126.7, 120.2, 82.5, 41.3, 40.2, 25.8, 18.3, 10.3, 9.6, -4.3, -4.3; **IR** ν_{max} (film): 2928 (br.), 1762, 1609, 1509, 1249, 1175, 1050, 903, 829 cm^{-1}. **HRMS** (ESI) calculated $[C_{18}H_{28}NaO_3Si]^+$ 343.1705, found 343.1701. **6b** (minor isomer): **^{1}H NMR** (400 MHz, $CDCl_3$) δ 7.17 (d, J = 8.5 Hz, 2H), 6.83 (d, J = 8.5 Hz, 2H), 4.98 (d, J = 6.7 Hz, 1H), 2.77 (qt, J = 7.6 Hz, 1H), 2.57-2.47 (m, 1H), 1.21 (d, J = 7.6 Hz, 3H), 1.06 (d, J = 7.0 Hz, 3H), 0.98 (s, 9H), 0.19 (s, 6H); **^{13}C NMR** (150 MHz, $CDCl_3$) δ 179.9, 156.0, 130.9, 127.1, 120.3, 85.9, 42.2, 38.5, 25.8, 18.3, 12.6, 10.4, -4.3, -4.3; **IR** ν_{max} (film): 2931, 1777, 1610, 1513, 1265, 1200, 994, 913, 839; **HRMS** (ESI) calculated $[C_{18}H_{28}NaO_3Si]^+$ 343.1705, found 343.1700.

5-(4-((*tert*-Butyldimethylsilyl)oxy)phenyl)-3,4-dimethyltetrahydrofuran-2-ol 7

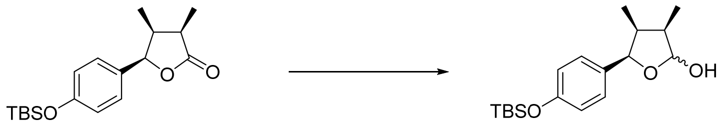

To a solution of lactone **6a** (1.282 g, 4.0 mmol) in dry CH_2Cl_2 (20 mL) at -78 °C DIBAL-H (1.0 M in heptane, 4.20 mL) was added dropwise under an argon atmosphere. After one hour at -78 °C, 2 mL methanol and 20 mL diethyl ether were added and the reaction mixture was allowed to warm up. At ca. -10 °C, the mixture was poured into 80 mL of a vigorously stirred solution of Rochelle's salt (20% in water). After addition of diethyl ether (30 mL) the layers were separated and the aqueous phase was extracted with diethyl ether (2 x 20 mL), washed with brine, dried over magnesium sulfate, and the filtrate concentrated in *vacuo*. Purification by column chromatography gave 1.278 g (99 %) of an inseparable diasteromeric mixture (4:1) of lactol **7** as a colorless liquid which solidified in the refrigerator overnight. **^{1}H NMR** (400MHz, $CDCl_3$) δ 7.12.(d, J = 8.4 Hz, 2H), 6.80 (d, J = 8.4 Hz, 2H), 5.35 (d, 4.7 Hz, 1H), 5.27 (d, J = 4.0 Hz, 1H), 2.97 (d, J = 4.0 Hz, 1H), 2.45-2.35 (m, 2H), 1.08 (d, J = 7.0 Hz, 3H), 0.97 (s, 9H), 0.50 (d, J = 7.2 Hz, 3H), 0.18 (s, 6H). **^{13}C NMR** (100 MHz, $CDCl_3$) δ 154.7, 132.2, 127.1, 119.8, 104.0, 83.2, 46.1, 42.0, 25.8, 18.3, 12.4, 10.0,-4.3, -4.3; **IR** ν_{max} (film): 3408 (br), 2928, 2856, 1611, 1509, 1462, 1351, 1255, 1167, 1066, 1027, 990, 902, 837; **HRMS** (ESI) calculated $[C_{18}H_{30}NaO_3Si]^+$ 345.1856, found 345.1858.

tert-Butyl(4-(-3,4-dimethyl-5-(phenylsulfonyl)tetrahydrofuran-2-yl)phenoxy)dimethylsilane 11

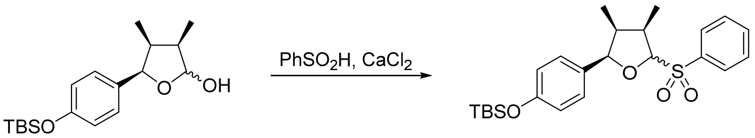

The reagent (Phenylsulfinic acid) is sensitive to oxidation in air and has to be freshly prepared. For this purpose, sodium phenylsulfinate (1.31 g, 8 mmol) was dissolved in aqueous sulfuric acid (2M, 30 mL) and stirred under argon for 30 minutes. Then DCM was added (30 mL) and stirring was continued for ten minutes. After separation of the layers, the aqueous phase was extracted with air-free DCM (2x20 mL), dried over magnesium sulfate and concentrated in *vacuo* until the acid precipitated. At this point, 50 mL heptane was added and the resulting white suspension was filtered under argon flow. Evaporation of solvents afforded 831 mg (73 %) of phenylsulfinic acid as a white solid which was kept under argon until usage. To a stirred solution of hemiacetal **7** (323 mg, 1.0 mmol) in DCM (5 mL) $PhSO_2H$ (570 mg, 4.0 mmol) and $CaCl_2$ (222 mg, 2 mmol) were added at room temperature. After stirring for 3 h, the reaction was quenched with aqueous $NaHCO_3$ (50 mL) and extracted with EtOAc (2x 20 mL). The combined organic layers were washed with brine, dried over $MgSO_4$ and concentrated in *vacuo*. Purification by column chromatography (Heptane/EtOAc = 5:1) afforded 310 mg of sulfone acetal **11** (ca. 90 % as a > 9:1 mixture of isomers with some traces of other impurities) which further decomposed during standing and was therefore directly used for the next step. ^{1}**H NMR** (600 MHz, $CDCl_3$) δ 7.97 (d, J = 7.5 Hz, 2H) 7.65 (t, J = 7.5 Hz,1H), 7.55 (t, 7.5 Hz, 2H), 7.01 (d, J = 8.0 Hz, 2H), 6.77 (d, J = 8.0 Hz, 2H), 5.31 (d, J = 4.5 Hz, 1H), 4.61 (d, J = 8.0 Hz, 1H), 3.27 (m, 1H), 1.30 (d, J = 7.0 Hz, 3H), 0.96 (s, 9H), 0.50 (d, J = 7.0 Hz, 3H), 0.17 (s, 6H); ^{13}**C NMR** (150 MHz, $CDCl_3$) δ 154.9, 137.6, 133.9, 129.5, 127.0, 119.8, 97.9, 86.0, 42.4, 38.7, 25.8, 18.3, 14.6, 9.5, -4.3; **IR** ν_{max} (film): 2930, 2858, 1610, 1512, 1447, 1318, 1260, 1149, 1061, 914, 838; **HRMS** (ESI) calculated for $[C_{30}H_{48}NaO_3Si_2]^+$ 4.69.1839, found 469.1835.

(((-3,4-Dimethyltetrahydrofuran-2,5-diyl)bis(4,1-phenylene))bis(oxy))bis(_tert_-butyldimethylsilane) 10

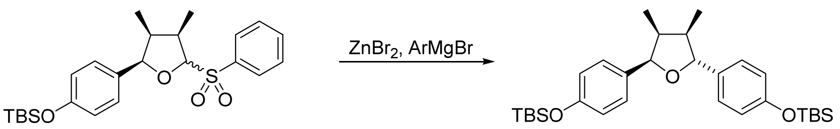

A solution of Grignard reagent (6.4 mL, 0.5 M in THF) was prepared according to the procedure above and was added to a freshly prepared solution of $ZnBr_2$ (360 mg, 1.6 mmol) in dry THF (16 mL) at room temperature. After 30 min, the solution was heated to 45 °C and kept at this temperature for 30 min. After cooling to 20 °C, a solution of sulfone **11** (179 mg, 0.40 mmol in 2 mL THF) was added and stirred for 0.5 h at room temperature and 1 h under refluxing conditions. The reaction was quenched with aqueous NH_4Cl (20 mL). After addition of diethyl ether (25 mL) and separation of the layers the aqueous phase was extracted with diethyl ether (2x 20 mL). The combined organic phases were dried ($MgSO_4$) and concentrated in *vacuo*. Purification by column chromatography (Heptane/EtOAc 40:1) gave **10** (182 mg, 88%). R_f: 0.16 (Heptane/EtOAc = 40:1). ^{1}**H NMR** (700 MHz, $CDCl_3$) δ 7.24 (d, J = 8.5 Hz, 2H), 7.19 (d, J = 8.5 Hz, 2H), 6.82-6.79 (m, 4H), 5.44 (d, J = 4.2 Hz, 1H), 4.63 (d, J = 9.2 Hz, 1H), 2.46-2.39 (m, 2H), 0.98-0.96 (d, J = 3 Hz, 3H overlain), (s, 18H), 0.60 (d, J = 7.0 Hz, 3H), 0.18 (s, 12H); ^{13}**C NMR** (175 MHz, $CDCl_3$) δ 155.1, 154.5, 136.0, 133.5, 127.4, 127.3, 120.1, 119.8, 85.6, 84.9, 47.8, 43.5, 25.9, 25.9, 18.4, 18.4, 11.9, 9.6, -4.3, -4.3, -4.3, -4.3; **IR** ν_{max} (film): 2927, 2856, 1609, 1509, 1462, 1253, 1064, 1009, 915, 837, 802; **HRMS (**ESI) calculated for $[C_{24}H_{34}NaO_4SSi]^+$ 535.3040, found 535.3037.

4,4'-(3,4-Dimethyltetrahydrofuran-2,5-diyl)diphenol (_rac_-Larreatricin) 1

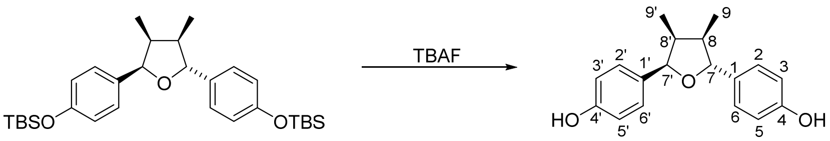

To a solution of **10** (180 mg, 0.36 mmol) in THF (5 mL) TBAF (1M in THF, 1.1 mL, 3 equiv) was added and stirred for 3 h. The reaction was worked up by addition of aqueous NH_4Cl (10 mL) and extraction with Et_2O (3x 10 mL). The combined organic layers were dried over $MgSO_4$ and concentrated in *vacuo*. Column chromatography (Heptane/EtOAc 2:1) afforded pure *rac*-larreatricin (102 mg, 94%) as colorless needles (m.p 160-162 °C, EtOAc). Separation of enantiomers was performed by chiral HPLC (Chiralpak IC, 48% hepatane, 50% BTBE, 2% EtOH) to give the (–)-enantiomer after 6.1 min and the (+)-enantiomer after 7.9 min. R_f: 0.27 (Heptane/EtOAc = 1:1). ^{1}**H NMR** (700 MHz, acetone-d_6) δ 8.33 (s_{br}, 1H), 8.27 (s_{br}, 1H), 7.27-7.24 (m, 2H), 6.85–6.81 (m, 4H), 5.43 (d, J = 4.6 Hz, 1H), 4.61 (d, J = 9.7 Hz, 1H), 2.49-2.38 (m, 2H), 0.98 (d, J = 6.8 Hz, 3H), 0.59 (d, J = 7.2 Hz, 3H); ^{13}**C NMR** (175 MHz) δ = 157.60, 157.01, 135.33, 132.72, 128.25, 127.93, 115.80, 115.48, 86.11, 85.16, 48.44, 44.01, 12.03, 9.74. **IR** ν_{max} (film): 3506(br),

3234(br), 2963, 2901, 1613, 1598, 1516, 1461, 1400, 1235, 1103, 1060, 961,830,817; **HRMS** (ESI) calculated for $[C_{18}H_{20}NaO_3]^+$ 307.1310, found 307.1308.

Table S1. Comparison of ^{13}C NMR data of synthetic larreatricin in acetone-d_6.

carbon #	**4** or **4'**		**1** or **1'**		**2/6** or **2'/6'**		**3/5** or **3'/5'**		**7** or **7'**		**8** or **8'**		**9** or **9'**	
this work	157.60	157.01	135.33	132.72	128.25	127.93	115.80	115.48	86.11	85.16	48.44	44.01	12.03	9.74
Lewis2003 [4]	157.6	157.6	135.5	132.8	128.3	127.9	115.8	115.5	86.1	85.2	48.4	44.0	12.1	9.7

3. Experimental Procedures for the enantioselective Synthesis of Lactone 6a

4-((tert-butyldimethylsilyl)oxy)phenyl)-zinc triflate

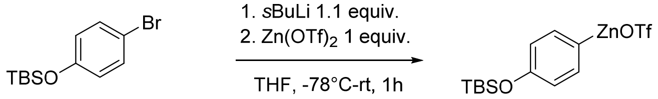

According to a slightly modified literature procedure.[5] A flame-dried Schlenk flask was purged with argon and was charged with aryl bromide **11** (287 mg, 1.0 mmol) and 0.7 mL dry THF. The solution was cooled to -78° C. To this, a solution of *sec*-BuLi (1.4 M in cyclohexane, 0.8 mL, 1.1 mmol) was added dropwise and the mixture was allowed to stir for 15 min. Meanwhile, a second flame-dried Schlenk flask was charged with $Zn(OTf)_2$ (364 mg, 1 mmol) in a glovebox. Upon removal from the glovebox, the solid was suspended in 0.5 mL dry THF. The aryl lithium was then added to the suspension of $Zn(OTf)_2$ at -78° C. The mixture was warmed up to room temperature and stirred for 1 hour to afford the desired organozinc triflate (0.5 M in THF).

(2*R*,3*S*)-4-(4-((tert-butyldimethylsilyl)oxy)phenyl)-2,3-dimethyl-4-oxobutanoic acid (+)-12

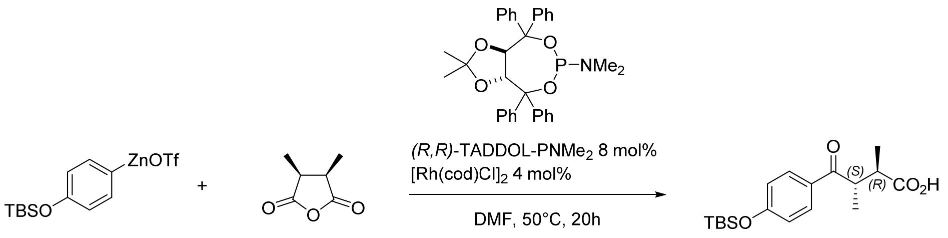

After a slightly modified literature procedure.[5] A flame-dried Schlenk flask was charged with $[Rh(cod)Cl]_2$ (3.9 mg, 0.008 mmol) and *(R,R)*-TADDOL-$PNMe_2$ (8.6 mg, 0.016 mmol). The flask was purged with argon and 1 mL dry DMF was added. The solution was stirred for 5 minutes before addition of the arylzinc triflate. The reaction mixture was heated to 50°C in an oil bath the anhydride in 0.5 mL DMF was added. After 20 hours, the reaction mixture was diluted with MTBE and quenched with 1M HCl. The layers were separated and the aqueous layer was extracted with MTBE (3 x 10 mL). The combined organic layers were dried on $MgSO_4$, filtered and concentrated in *vacuo*. The crude product was purified on column chromatography (Heptane/EtOAc = 4:1 to 3:1) to yield the desired ketoacid **12** (26 mg, 0.077 mmol, 38%). R_f: 0.25 (Heptane/EtOAc = 1:1). **^{1}H NMR** (400 MHz, $CDCl_3$) δ 10.27 (br.s, 1H), 7.92 (m, 2H), 6.90 (m, 2H), 3.66 (p, J = 7.2 Hz, 1H), 2.98 (p, J = 7.0 Hz, 1H), 1.26 (d, J = 7.2 Hz, 3H), 1.21 (d, J = 7.0 Hz, 3H), 0.99 (s, 9H), 0.25 (s, 6H); **^{13}C NMR** (100 MHz) δ 202.5, 178.6, 161.1, 131.0, 129.6, 120.3, 43.3, 42.2, 25.7, 18.4, 16.5, 16.4, -4.2. **IR** ν_{max} (film): 2955, 2930, 2884, 2858, 1707, 1674, 1595, 1572, 1507, 1462, 1414, 1379, 1361, 1259, 1211, 1161, 1105, 1077, 1008, 976, 908, 837, 807, 781, 732, 714; **HRMS** (ESI) calculated for $[C_{18}H_{28}NaO_4Si]^+$ 359.1649, found 359.1646. $[\alpha]_D^{23}$ = +11.8° (c = 1.0, $CHCl_3$). ***ee*** = 89 % (obtained from the corresponding methyl ester, see below).

(2*R*,3*S*)-Methyl 4-(4-((tert-butyldimethylsilyl)oxy)phenyl)-2,3-dimethyl-4-oxobutanoate (-)-**13**

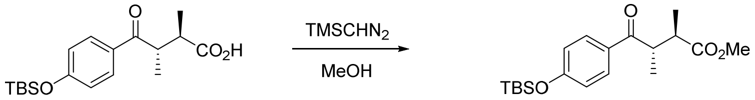

The ketoacid **12** (26 mg, 0.078 mmol) was dissolved in 1 mL MeOH and cooled to 0 °C. TMS-diazomethane (2.0M in Et_2O, 0.1 mL) was added dropwise and the reaction was stirred for 30 minutes at 0 °C. The reaction was quenched with a few drops of AcOH until the yellow color disappeared and EtOAc (5 mL) and 1M HCl (3 mL) were added. The layers were separated and the aqueous layer was extracted with EtOAc (2 x 5 mL). The combined organic layers were dried on $MgSO_4$, filtered and evaporated in *vacuo* to afford the pure methyl ester (26 mg, 0.076 mmol, 97%). **^{1}H NMR** (400 MHz, $CDCl_3$) δ 7.91 (d, J = 8.5 Hz, 2H), 6.89 (d, J = 8.5 Hz, 2H), 3.71 (m, 3H), 3.67 (m, 1H), 2.95 (m, 1H), 1.16 (d, J = 6.9 Hz, 3H), 1.11 (d, J = 6.9 Hz, 3H), 0.24 (s, 6H), 0.99 (s, 9H); **^{13}C NMR** (100 MHz) δ 201.3, 176.4, 160.6, 130.7, 130.4, 120.2, 51.8, 43.4, 43.0, 25.7, 16.4, 18.4, 17.1, -4.2. **IR** ν_{max} (film): 2953, 2930, 2858, 1736, 1674, 1596, 1506, 1461, 1256, 1215, 1192, 1161, 1105, 1076, 1059, 976, 907, 838, 824, 808. **HRMS** (ESI) calculated for $[C_{18}H_{28}NaO_4Si]^+$ 373.1806, found 373.1803. **$[\alpha]_D^{20}$** = -29.5° (c = 1.0, $CHCl_3$). Enantiomeric ratio 95.5:4.5 (***ee*** = 89%) was determined by chiral HPLC analysis (Chiralpak IC column, 98:2 hept/iPrOH, 1.0 mL/min, 210 nm, (tr major) = 9.8 min, tr (minor) = 10.7).

(3*R*,4*S*,5*R*)-5-(4-((tert-butyldimethylsilyl)oxy)phenyl)-3,4-dimethyldihydrofuran-2(3H)-one (+)-**6a**

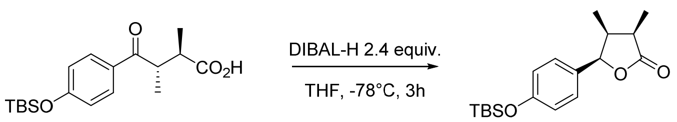

A flame-dried Schlenk with ketoacid **12** (26 mg, 0.077 mmol) in 1 mL dry THF was cooled to -78°C. Diisobutylaluminium hydride (0.19 ml, 1M in THF, 2.4 equiv.) was added dropwise and the reaction was stirred for 3h at -78°C. The reaction was quenched with 1M HCl and warmed up to room temperature. The layers were separated and the aqueous layer was extracted with EtOAc (2x 15 mL). The combined organic layers were dried on $MgSO_4$, filtered and evaporated in *vacuo* to afford the pure product as a mixture of diastereomers (6.2:1 d.r.). The diastereomers were separated on column chromatography (Heptane/MTBE = 4:1) to afford the desired product (18 mg, 0.056 mmol, 73%). Characterization data for racemic **6a** see above. **$[\alpha]_D^{23}$** = +32.8° (c = 1.0, $CHCl_3$). Enantiomeric ratio 93:7 (***ee*** = 86 %) was determined by chiral HPLC analysis (Lux-3 Cellulose-3 column, 99:0.5:0.5 hept/EtOH/^{i}PrOH, 0.7 mL/min, 210 nm, retention time (min): 11.0 (major) and 12.6 (minor).

Screening of conditions for the diastereoselective reduction of keto-acid 10.

Table S2. Diastereoselective reduction and ring-closure (reductants and additives).

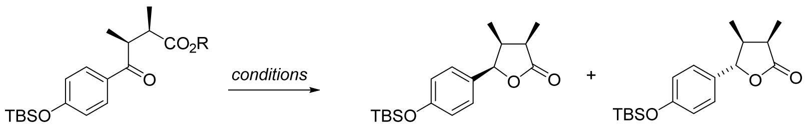

Entry[a]	R	Solvent	Reductant(equiv.)	Additive [b]	**6a:6b**[c]
1	Me	THF/H_2O 9:1	$NaBH_4$ (1)	-	1:18
2	Me	THF/H_2O 9:1	$NaBH_4$ (1)	$MgBr_2$	1:18
3	H	THF/H_2O 9:1	$NaBH_4$ (1)	-	1:5
4	H	THF/H_2O 9:1	$NaBH_4$ (1)	$MgBr_2$	2:1
5	H	THF/H_2O 1:1	$NaBH_4$ (1)	$MgBr_2$	1:1.4
6	H	THF/H_2O 99:1	$NaBH_4$ (1)	$MgBr_2$	2:1
7	H	EtOH	$NaBH_4$ (1)	$MgBr_2$	2.6:1
8	H	THF/H_2O 9:1	$NaBH_4$ (1)	$MgCl_2$	1:1.5
9	H	THF/H_2O 9:1	$NaBH_4$ (1)	MgI_2	1:4
10	H	THF/H_2O 9:1	$NaBH_4$ (1)	$Mg(OTf)_2$	1:3.8
11	H	THF/H_2O 9:1	$NaBH_4$ (1)	$Zn(OTf)_2$	1:3.6
12	H	THF/H_2O 9:1	$NaBH_4$ (1)	iPrMgCl	1:5.6
13	H	THF/H_2O 9:1	$NaBH_4$ (1)	$CeCl_3$	1:3.2
14	H	THF/H_2O 9:1	$NaBH_4$ (1)	$CaCO_3$	1:2.7
**15[d]**	**H**	**THF**	**DIBAL-H (2.4)**	**-**	**6.2:1**
16[d]	H	Toluene	DIBAL-H (2.4)	-	1:9.8
17[d]	H	THF	DIBAL-H (2.4)	$ZnCl_2$	1:2.1

[a] Reaction was performed at 0 °C, unless otherwise stated. [b] 1 equivalent of additive. [c] Ratio was determined by ^{1}H-NMR analysis of the crude. [d] Reactions were performed at -78°C.

4. Selected NMR Spectra

Key lactone **6a** and its diastereomer **6b**

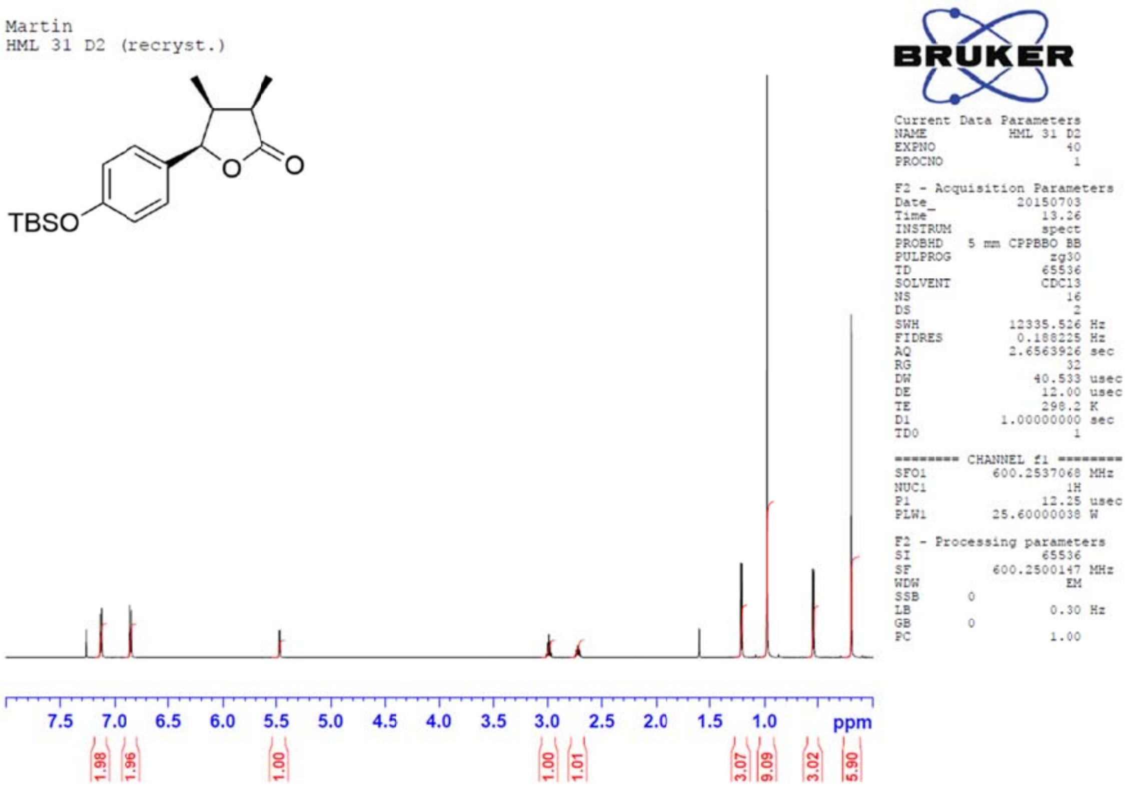

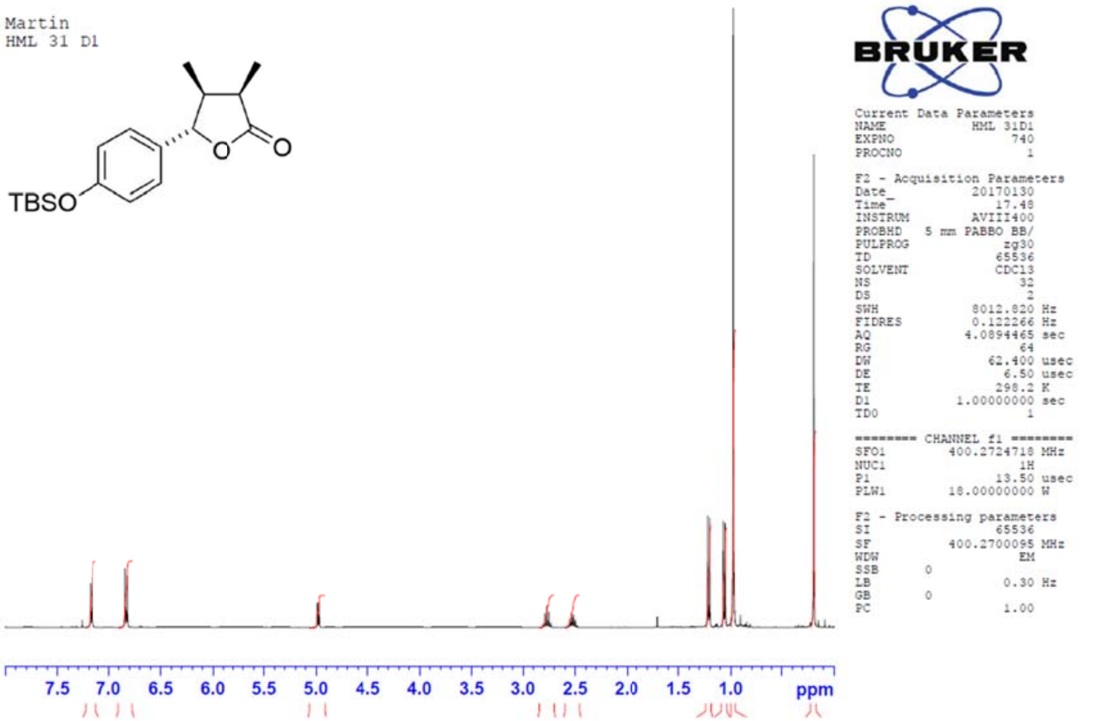

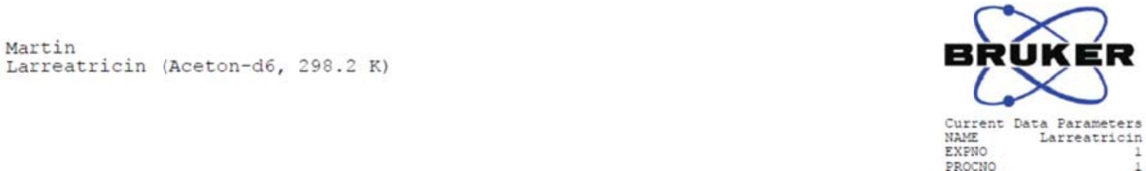

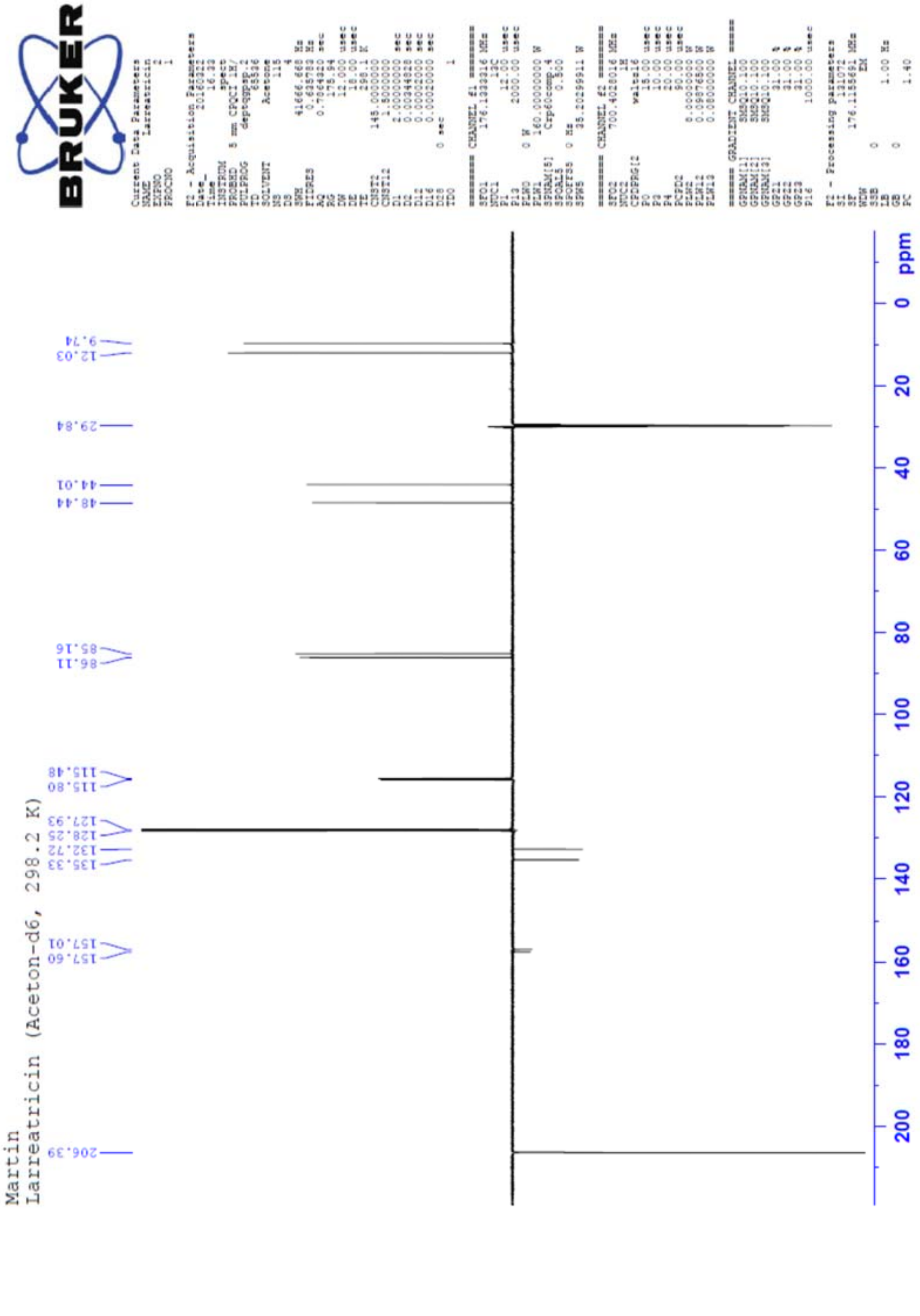
Martin
Larreatricin (Aceton-d6, 298.2 K)
9.74
12.03
29.84
44.01
48.44
85.16
86.11
115.48
115.80
127.93
128.25
132.72
135.33
157.01
157.60
206.39
ppm
0
20
40
60
80
100
120
140
160
180
200

5. HPLC Reports

Larreatricin *rac*-**1** (6.11 min, 7.90 min) and (-)-**1** (6.33 min)

Sample Information

Sample Name	: HML44 Racemat _IC_2Inj
Sample ID	:
Vial#	: 1
Injection Volume	: 12
Data File	: HML44_IC_2Inj_04.03.2016_1_001.lcd
Method File	: Run_ISOHeptan75-IPA25_F1_Pos2.lcm
Batch File	: 04.03.2016_1.lcb
Report Format File	: REPORTChiralpak ICHep75_IPA25F1ml.lsr
Date Acquired	: 04.03.2016 11:59:39
Date Processed	: 04.03.2016 12:13:47

Detector A Channel 1 210nm

Peak#	Ret. Time	Area	Area%
1	6,114	6337000	50,089
2	7,899	6314405	49,911
Total		12651405	100,000

Detector A Channel 2 230nm

Peak#	Ret. Time	Area	Area%
1	6,116	12503539	50,040
2	7,900	12483727	49,960
Total		24987266	100,000

Method Description:
Column:Chiralpak IC 250x4,6mm Particle Size 5 micrometer
Solvent System: n-Heptan+0,1%IPA/IPA 75:25
Flow:1 ml/min

Chromatogram

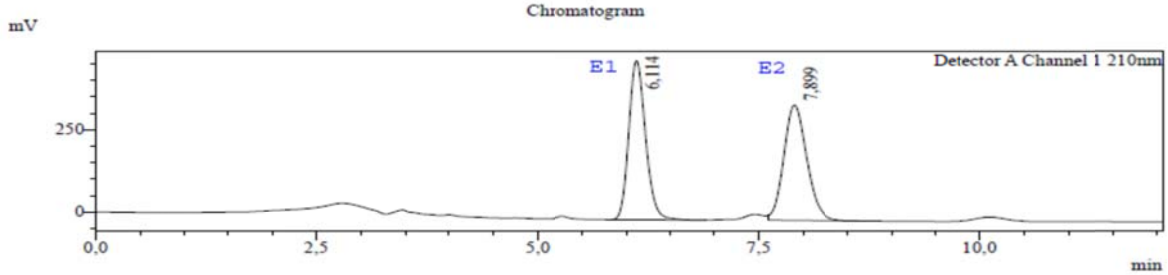

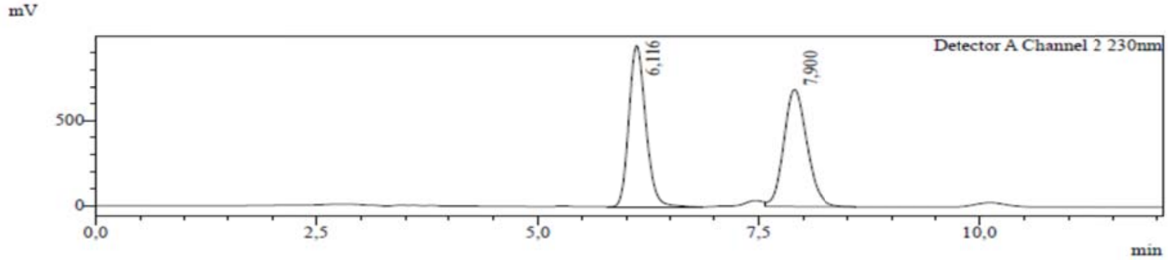

Sample Information

Sample Name	: HML44_E1_nachPrep_2Inj
Sample ID	:
Vial#	: 18
Injection Volume	: 12
Data File	: HML44_E1_nachPrep_2Inj_17.03.2016_1_001.lcd
Method File	: Run_ISOHeptan75-IPA25_F1_Pos2.lcm
Batch File	: 17.03.2016_1.lcb
Report Format File	: REPORTChiralpak ICHep75_IPA25F1ml.lsr
Date Acquired	: 17.03.2016 12:36:12
Date Processed	: 27.04.2016 11:36:01

Detector A Channel 1 210nm

Peak#	Ret. Time	Area	Area%
1	6,326	5561317	100,000
Total		5561317	100,000

Detector A Channel 2 230nm

Peak#	Ret. Time	Area	Area%
1	6,328	10987521	100,000
Total		10987521	100,000

Method Description:
Column:Chiralpak IC 250x4,6mm Particle Size 5 micrometer
Solvent System: n-Heptan+0,1%IPA/IPA 75:25
Flow:1 ml/min

Chromatogram

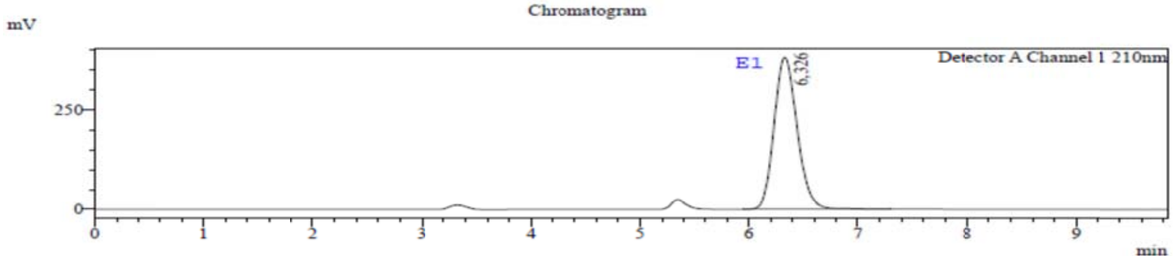

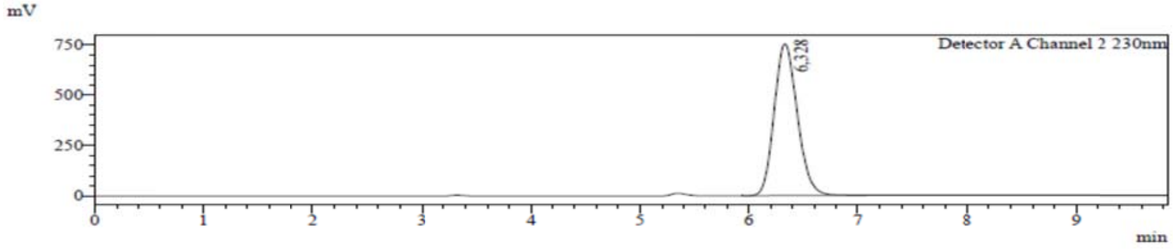

Ketoester *rac*-**13** (9.79 min, 10.77 min) and (-)-**13** (9.78 min)

Sample Information

Sample Name : RIOO1510Rac_IC
Sample ID :
Vial# : 16
Injection Volume : 6
Data File : RIOO1510Rac_IC_25.10.2017_1_005.lcd
Method File : Run_ISOHeptan98-IPA2_F1_Pos2.lcm
Batch File : 25.10.2017_1.lcb
Report Format File : REPORTChiralpak ICHep9,8_IPA0,2F1.lsr
Date Acquired : 25.10.2017 13:51:23
Date Processed : 25.10.2017 14:21:25

Method Description:
Column:Chiralpak IC 250x4,6mm Particle Size 5 micrometer
Solvent System: n-Heptan+0,1%IPA/IPA 9,8:0,2
Flow:1 ml/min T=25°C

Detector A Channel 1 210nm

Peak#	Ret. Time	Area	Area%
1	9,785	3747267	50,464
2	10,773	3678359	49,536
Total		7425626	100,000

Detector A Channel 2 254nm

Peak#	Ret. Time	Area	Area%
1	9,787	3913862	50,076
2	10,774	3901988	49,924
Total		7815850	100,000

Chromatogram

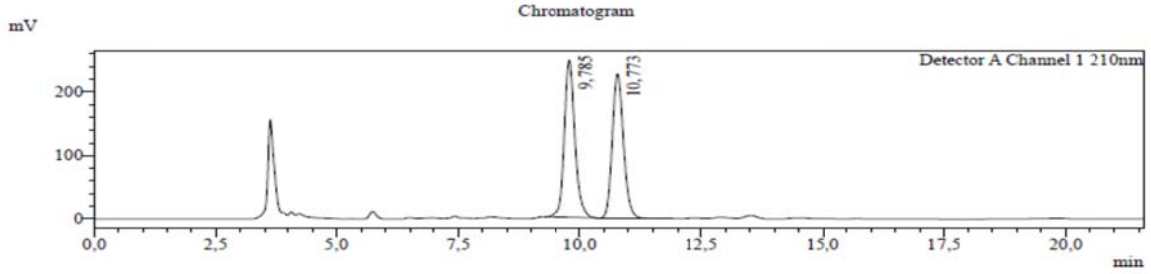

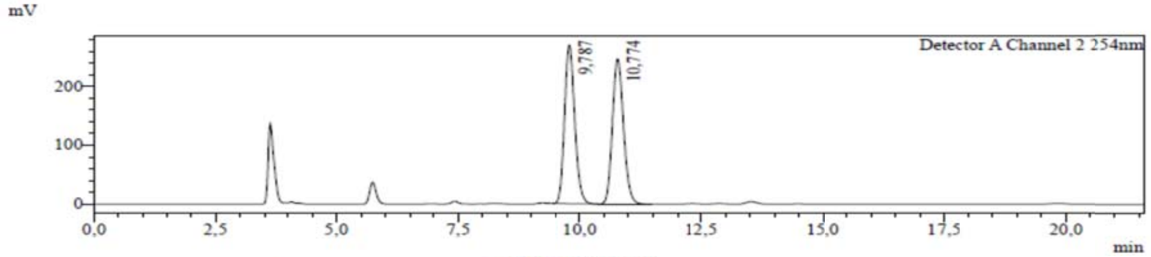

Sample Information

Sample Name : RIOO1517_1Inj
Sample ID :
Vial# : 77
Injection Volume : 6
Data File : RIOO1517_1Inj_06.11.2017_3_006.lcd
Method File : Run_ISOHeptan98-IPA2_F1_Pos2.lcm
Batch File : 06.11.2017_3.lcb
Report Format File : REPORTChiralpak ICHep9,8_IPA0,2F1.lsr
Date Acquired : 07.11.2017 01:19:08
Date Processed : 07.11.2017 09:48:42

Method Description:
Column:Chiralpak IC 250x4,6mm Particle Size 5 micrometer
Solvent System: n-Heptan+0,1%IPA/IPA 9,8:0,2
Flow:1 ml/min T=25°C

Detector A Channel 1 210nm

Peak#	Ret. Time	Area	Area%
1	9,784	10243426	94,575
2	10,791	587576	5,425
Total		10831002	100,000

Detector A Channel 2 254nm

Peak#	Ret. Time	Area	Area%
1	9,785	11041151	94,427
2	10,793	651657	5,573
Total		11692808	100,000

Chromatogram

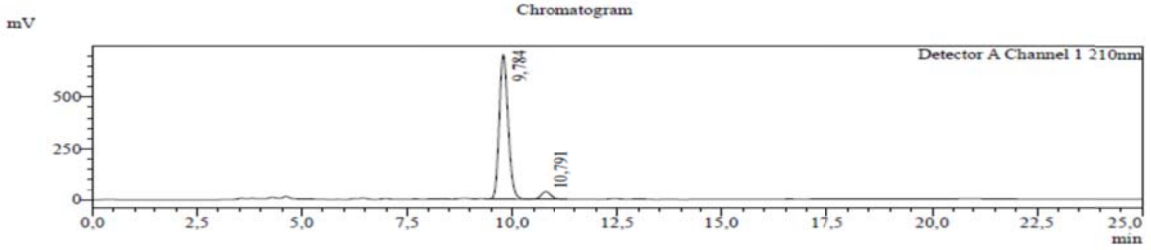

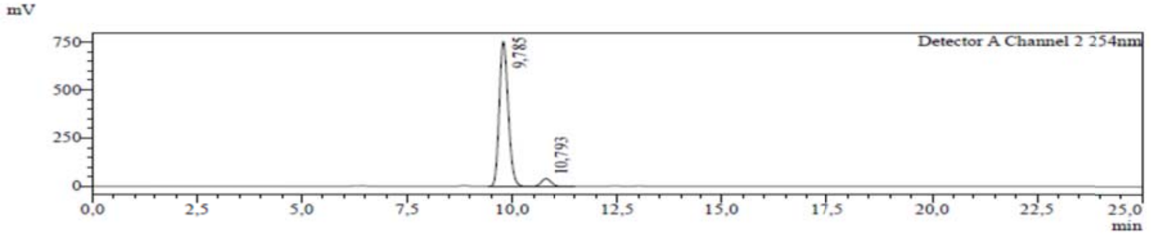

Lactone *rac*-**6a** (10.99 min, 12.63 min) and (+)-**6a** (10.99 min)

Sample Information

Sample Name	: HML31D2Rac_Lux_3
Sample ID	:
Vial#	: 92
Injection Volume	: 6
Data File	: HML31D2Rac_Lux_3_20.10.2017_1_001.lcd
Method File	: Run_100%D_F0,7_210nm.lcm
Batch File	: 20.10.2017_1.lcb
Report Format File	: REPORTLux Hep99_EtOH0,5_IPA0,5_F0,7.lsr
Date Acquired	: 20.10.2017 12:48:54
Date Processed	: 20.10.2017 15:31:12

Detector A Channel 1 210nm

Peak#	Ret. Time	Area	Area%
1	10,992	17650549	50,065
2	12,629	17604689	49,935
Total		35255238	100,000

Detector A Channel 2 254nm

Peak#	Ret. Time	Area	Area%
1	10,993	817088	50,093
2	12,631	814062	49,907
Total		1631150	100,000

Method Description:
Column:Lux-3 Cellulose-3
250x4,6mm Particle Size 3 micrometer
Solvent System: n-Heptan/EtOH/IPA 99:0,5:0,5
Flow=0,7 ml/min T=25°C

Chromatogram

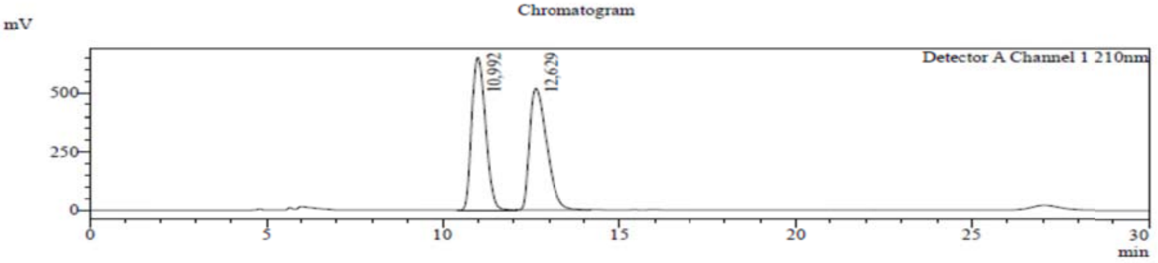

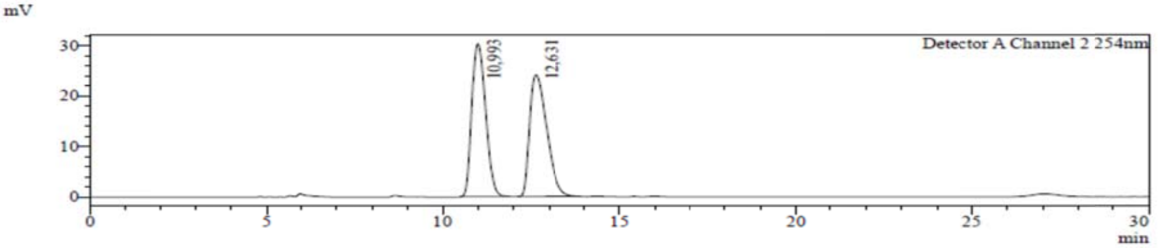

Sample Information

Sample Name	: RIOO1575_2Inj
Sample ID	:
Vial#	: 1
Injection Volume	: 6
Data File	: RIOO1575_2Inj_19.12.2017_1_001.lcd
Method File	: Run_100%D_F0,7.lcm
Batch File	: 19.12.2017_1.lcb
Report Format File	: REPORTLux Hep99_EtOH0,5_IPA0,5_F0,7.lsr
Date Acquired	: 19.12.2017 12:07:15
Date Processed	: 19.12.2017 14:40:08

Detector A Channel 1 210nm

Peak#	Ret. Time	Area	Area%
1	10,984	8211456	93,176
2	12,569	601425	6,824
Total		8812881	100,000

Detector A Channel 2 230nm

Peak#	Ret. Time	Area	Area%
1	10,986	11445839	92,723
2	12,571	898288	7,277
Total		12344126	100,000

Method Description:
Column:Lux-3 Cellulose-3
250x4,6mm Particle Size 3 micrometer
Solvent System: n-Heptan/EtOH/IPA 99:0,5:0,5
Flow=0,7 ml/min T=25°C

Chromatogram

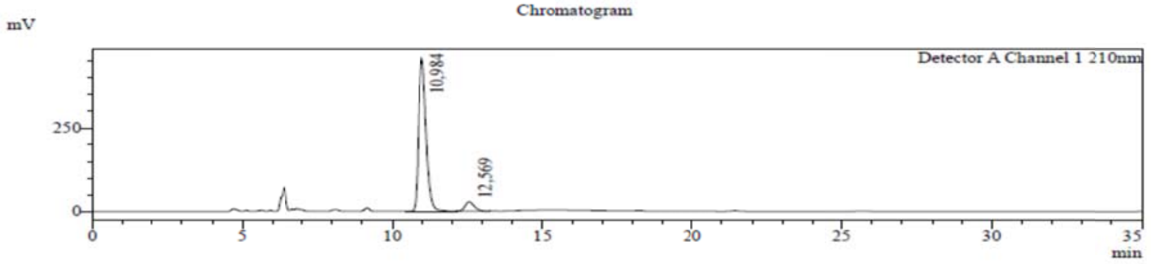

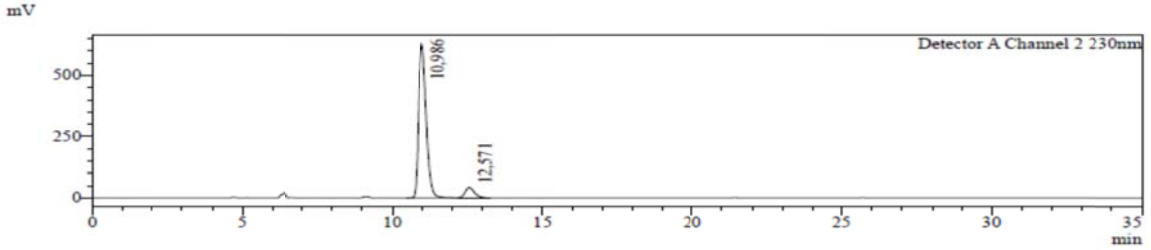

6. X-Ray Crystallographic Data

The X-ray intensity data was measured on a Bruker X8 Apex2 diffractometer equipped with multilayer monochromators, Mo K/α INCOATEC micro focus sealed tubes and Kryoflex cooling devices. The structure was solved by direct methods and refined by full-matrix least-squares techniques. Non-hydrogen atoms were refined with anisotropic displacement parameters. Hydrogen atoms were inserted at calculated positions and refined with a riding model. The following software was used: Frame integration, *Bruker SAINT software package*[6] using a narrow-frame algorithm, Absorption correction, *SADABS*[7], structure solution, *SHELXS-2015*[8], refinement, *SHELXL-2015*[8], *OLEX2*[9], *SHELXLE*[10], molecular diagrams and structural illustrations, *OLEX2*[9] and *Crystal Diamond*[11]. Experimental data and CCDC-code can be found in **Table S3**. Crystal data, data collection parameters, and structure refinement details are given in **Tables S4** to **S7**. Molecular Structures are displayed in **Figures S1 to S3**.

Table S3. Experimental parameters and CCDC-Code.

Sample	Machine	Source	Temp.	Detector Distance	Time/ Frame	#Frames	Frame width	CCDC
			[K]	[mm]	[s]		[°]	
$C_{18}H_{28}O_3Si$	Bruker X8	Mo	150	35	6	2278	0.5	1823645
$C_{30}H_{48}O_3Si_2$	Bruker X8	Mo	100	35	60	1352	0.3	1823646

(3R*,4S*,5R*)-5-(4-((tert-butyldimethylsilyl)oxy)phenyl)-3,4-dimethyldihydrofuran-2(3H)-one 6a

The trisubstituted lactone **6a** crystallizes centrosymmetrically monoclinic in space group C 1 2/c 1 with the expected relative absolute configurational assignment, namely as (3R*,4S*,5R*)-5-(4-((tert-butyldimethylsilyl)oxy)phenyl)-3,4-dimethyldihydrofuran-2(3H)-one. C-bound H atoms were placed in idealized positions with C-H = 0.93 Å (aromatic CH), 0.96 Å (methyl CH_3) and 0.98 Å (furanyl CH) and refined with a riding model. TBSO (= tert-butyldimethylsilyl ether) at position C7A in the aromatic ring was the only disordered part in the molecule. Both methyl groups and the aryl group on the furan ring, which itself adopts a twisted conformation, arrange in the expected cis manner.

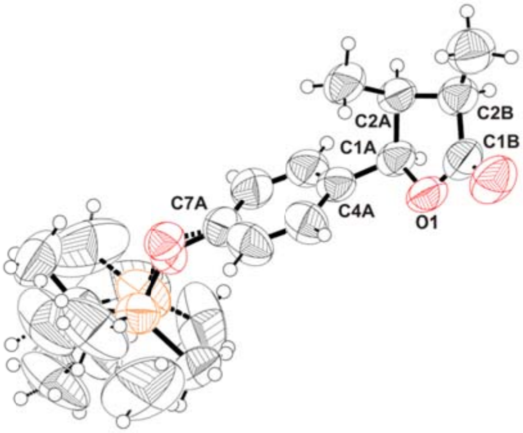

Figure S1. ORTEP-like view of (3R,4S,5R)-5-(4-((tert-butyldimethylsilyl)oxy)phenyl)-3,4-dimethyldihydrofuran-2(3H)-one (+)-**6a** with displacement ellipsoids for non-H atoms at the 50% probability level. Selected bond distances (Å) and angles (°), for **6a**: C1B-C2B 1.497, C2B-C2A 1.533, C2A-C1A 1.531, C1A-O1 1.464, O1-C1B 1.347, C2A-C1A-C4A 116.986 (222), O1-C1A-C4A 110.398(189).

Table S4. Sample and crystal data of **6a**.

**Chemical formula**	C18 H28 O3 Si	**Crystal system**	monoclinic	
**Formula weight [g/mol]**	320.49	**Space group**	*C 1 2/c 1*	
**Temperature [K]**	300	**Z**	8	
**Measurement method**	\Φ and \ω scans	**Volume [Å^3]**	3894.4(4)	
**Radiation (Wavelength [Å])**	MoKα (λ = 0.71073)	**Unit cell dimensions [Å] and [°]**	33.544(2)	90
**Crystal size / [mm^3]**	0.22 x 0.14 x 0.09		10.4181(5)	99.736(3)
**Crystal habit**	block		11.3067(8)	90
**Density (calculated) / [g/cm^3]**	1.093	**Absorption coefficient / [mm^{-1}]**	0.130	
**Abs. correction Tmin**	0.6487	**Abs. correction Tmax**	0.7460	
**Abs. correction type**	multi-scan	**F(000) [e$^-$]**	1392.0	

Table S5. Data collection and structure refinement of **6a**.

**Index ranges**	-40 ≤ h ≤ 40, -12 ≤ k ≤ 12, -13 ≤ l ≤ 13	**Theta range for data collection [°]**	4.1 to 50.7
**Reflections number**	39720	**Data / restraints / parameters**	3562/68/283

**Refinement method**	Least squares	**Final R indices**	all data	R1 = 0.1010, wR2 = 0.2193
**Function minimized**	Σ w(F_o^2 - F_c^2)^2		I>2σ(I)	R1 = 0.0608, wR2 = 0.1839
**Goodness-of-fit on F^2**	1.073	**Weighting scheme**	w=1/[δ^2(Fo^2)+(0.1018P)^2+1.6781P]	
**Largest diff. peak and hole [e Å^{-3}]**	0.19/-0.16		where P=(Fo^2+2Fc^2)/3	

(2S,3R,4S,5S)-2,5-bis(4-(tert-butyldimethylsilyl)oxy)-3,4-dimethyl tetrahydrofuran <u>10*</u>

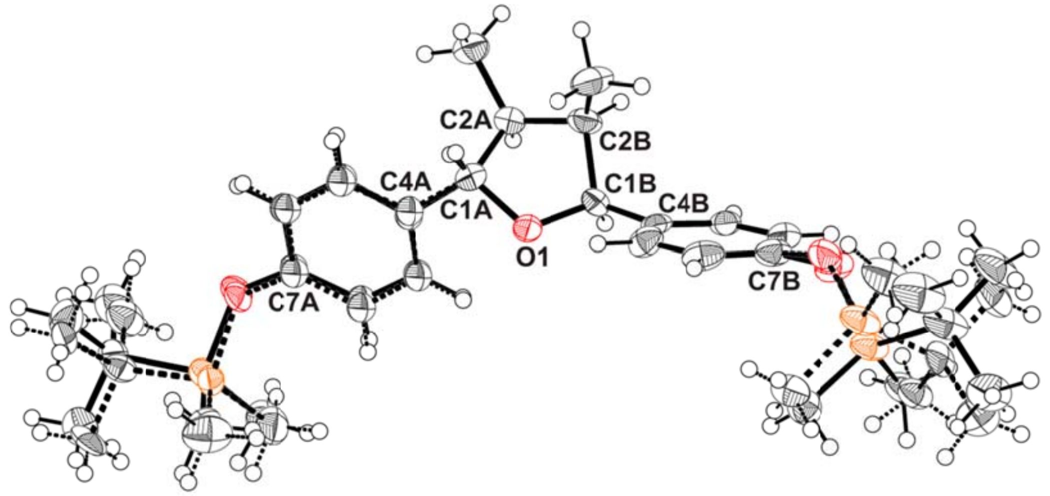

Figure S2. ORTEP-like view of silylated (-)-larreatricin with displacement ellipsoids for non-H atoms at the 50% probability level. Selected bond distances (Å) and angles (°), for **10***: C1A-O1 1.453, C1A-C2A 1.540, C2A-C2B 1.527, C2B-C1B 1.530, C1B-O1 1.433, O1-C1A-C4A 110.723(612), C2A-C1A-C4A 109.227(640), C2B-C1B-C4B 114.983(294), O1-C1B-C4B 112.351(327).

The corresponding *tert*.-butydimethylsilyl ether of (-)-larreatricin crystallizes monoclinic in the chiral space group P 1 2_1 1 with the expected relative absolute configurational assignment, namely as (2S,3R,4S,5S)-2,5-bis(4-(*tert*-butyldimethylsilyl)oxy)-3,4-dimethyl tetrahydrofuran. C-bound H atoms were placed in idealized positions with C-H = 0.95 Å (aromatic CH), 0.98 Å (methyl CH_3) and 0.98-1.00 Å (furanyl CH) and refined with a riding model. All four stereogenic centers on the twisted furan ring, both methyl groups and the aromatic part in the aryl group on C1B are not disordered, whereas the aromatic part of the opposite aryl residue bonded to C1A shows slight disorder. However, with sufficient probability, the three-dimensional structure of this compound can be confirmed.

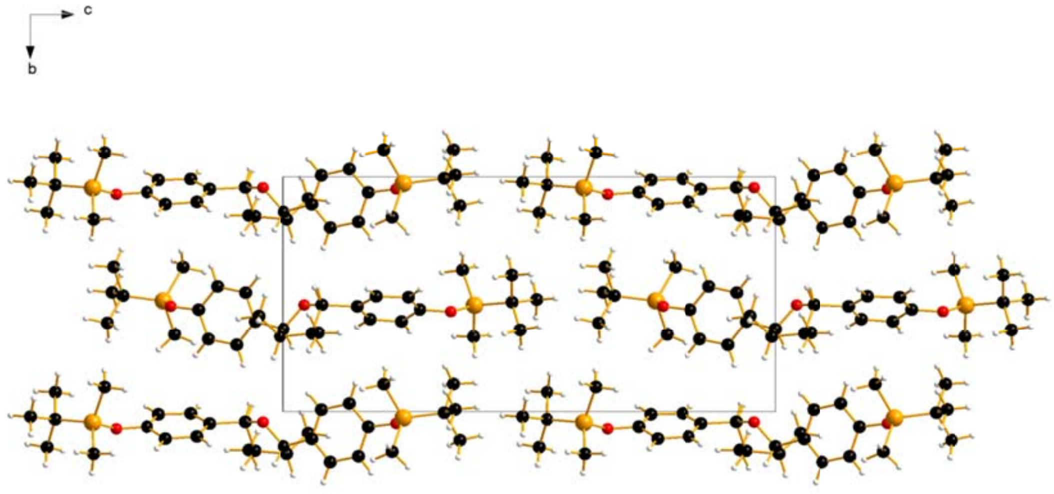

Figure S3. A detailed illustration of the crystal packing of **10*** along the b- and c-axis showing that the aryl residues at the chiral centers C1A (5S) and C1B (2S) are twisted by 90° to each other both in molecule and within the planes to the adjacent aryl residues.

Table S6. Sample and crystal data of **10***.

**Chemical formula**	$C_{30}H_{48}O_3Si_2$	**Crystal system**	monoclinic	
**Formula weight [g/mol]**	512.88	**Space group**	P 1 2_1 1	
**Temperature [K]**	100	**Z**	2	
**Measurement method**	\Φ and \ω scans	**Volume [Å^3]**	1538.5(3)	
**Radiation (Wavelength [Å])**	MoKα (λ = 0.71073)	**Unit cell dimensions [Å] and [°]**	6.7103(5)	90
**Crystal size / [mm^3]**	0.13 × 0.05 × 0.02		10.4605(12)	90.113(5)
**Crystal habit**	block		21.919(2)	90)
**Density (calculated) / [g/cm^3]**	1.1070	**Absorption coefficient / [mm^{-1}]**	0.142	
**Abs. correction Tmin**	0.6114	**Abs. correction Tmax**	0.7460	
**Abs. correction type**	multi-scan	**F(000) [e$^-$]**	560.5	

Table S7. Data collection and structure refinement of **10***.

**Index ranges**	-9 ≤ h ≤ 9, -14 ≤ k ≤ 14, -30 ≤ l ≤ 30	**Theta range for data collection [°]**	5.38 to 60.4	
**Reflections number**	15899	**Data / restraints / parameters**	8961/33/459	
**Refinement method**	Least squares	**Final R indices**	all data	R1 = 0.1654, wR2 = 0.1412
**Function minimized**	Σ w(F_o^2 - F_c^2)^2		I>2σ(I)	R1 = 0.0655, wR2 = 0.1100
**Goodness-of-fit on F^2**	0.982	**Weighting scheme**	w=1/[δ^2(Fo^2)+(0.0456P)^2]	
**Largest diff. peak and hole [e Å^{-3}]**	0.46/-0.42		where P=(Fo^2+2Fc^2)/3	

PART B Polyphenol Oxidase Production and Kinetic Characterization with Larreatricin Enantiomers

1. Plant Material, Cloning and Sequencing of Larreatricin Hydroxylase.

Young healthy leaves were obtained from *Larrea tridentata*, ground in liquid nitrogen, and total RNA was isolated by using the RNeasy® Plant Mini Kit (Qiagen, Hilden, Germany) according to the manufacturer's instructions. cDNA was synthesized using the SMARTer® RACE cDNA Amplification Kit (Clontech, Saint-Germain-en-Laye, France). Specific primers (Table S8) were designed to amplify the *Larrea tridentata* cDNA, guided by the sequence deposited in the ENA-GenBank-DDBJ: (+)-larreatricin hydroxylase (accession no. AY370019). PCR amplification from cDNA was done with Q5® High-Fidelity DNA polymerase (NEB, Ipswich, England). PCR products were cloned into the donor vector pENTRY-IBA51 (IBA Lifesciences, Göttingen, Germany) using the *Sap*I (NEB) recognition sequences and T4 DNA ligase (NEB). Once a positive colony containing the desired amplicon was obtained, the gene for larreatricin hydroxylase was subcloned into the expression vector pPSG-IBA25 with the use of *Esp*3I (Thermo Fisher scientific, Massachusetts, USA) and T4 DNA ligase (NEB). The expression vector pPSG-IBA25 containing the gene for larreatricin hydroxylase was transformed into chemically component *Escherichia coli* TOP10 cells (Thermo Fisher scientific). The clones were sequenced externally by Microsynth GmbH (Vienna, Austria). Sanger sequencing confirmed the open reading frames in the larreatricin hydroxylase clones. Sequence verified plasmids were transformed into ScarabXpress® T7lac *E. coli* (Scarab Genomics, Madison, USA) for subsequent heterologous expression.

2. Heterologous Expression and Purification of recombinant Larreatricin Hydroxylase.

Larreatricin hydroxylase was N-terminally fused with the GST-tag of the pPSG-IBA25 vector. Between the two fusion-partners the human rhinovirus 3C protease (HRV3C) recognition sequence (LEVLFQ|GP) is located which allows for the controlled dissociation of the two proteins. The fusion gene (GST-larreatricin hydroxylase) was efficiently overexpressed using the strong bacteriophage T7 promoter of the pPSG-IBA25 vector. *E. coli* was grown in LB medium (1 % tryptone-peptone, 0.5 % yeast extract and 1 % NaCl) supplemented with ampicillin (100 mg/l). Expression cultures were inoculated directly with a freshly transformed colony and were grown at 30 °C under shaking for 14 hours until the OD_{600} reached a value between 0.6 and 0.8. Afterwards, the temperature was reduced to 25 °C and the culture was induced with 0.5 mM isopropyl-β-D-1-thiogalactopyranoside and 0.5 mM $CuSO_4$. The expression cultures remained at 25 °C under shaking for 20 hours. After that time, the cultures were collected by centrifugation at 10000 x g for 25 minutes at 4 °C.

Lysis of the cells was effectuated by the freeze-thaw technique using liquid nitrogen. The pellets were resuspended in lysis buffer (50 mM Tris-HCl pH 7.5, 200 mM NaCl, 1 mM EDTA, 50 mM sucrose). Lysozyme (0.5 g/l) and protease inhibitors (1 mM phenylmethylsulfonyl fluoride and 1 mM benzamidine) were added and the resulting suspensions were incubated for 45 minutes under shaking on ice. Subsequently, the solutions underwent five cycles of freezing in liquid nitrogen and thawing in a water bath at 25 °C. Eventually, 2 mM $MgCl_2$ and 0.02 g/l DNaseI were added to the lysates and they were incubated for 15 minutes at 100 rpm and 25 °C. The lysates were centrifuged at 10000 x g for 1 hour at 4 °C.

Chromatographic purifications were carried out using an Äkta Purifier (GE Healthcare) placed in a refrigerator at 4 °C. The filtrated lysates were placed in a 50 ml injection loop and applied to a prepacked 5 ml GSTrap FF column using 50 mM Tris-HCl pH 7.5, 200 mM NaCl as the binding buffer. Following the trapping and flushing out of unbound proteins, the target proteins were eluted with 50 mM Tris-HCl pH 7.5, 200 mM NaCl and 15 mM reduced glutathione. The GST-fusion protein fraction was concentrated using a Vivaspin® ultrafiltration device with a 30 kDa molecular weight cut-off (VWR). The buffer was exchanged to 50 mM Tris-HCl pH 7.0, 150 mM NaCl, 1 mM EDTA and the samples were mixed with GST-HRV3C produced in-house according to [12] at a mass ratio of 1 to 40 (protease to fusion protein). The proteolysis was carried out over 48 hours at 4 °C. The cleaved protein was then again applied to a 5 ml GSTrap FF column, whereby the GST protein and the GST-tagged protease were still trapped by the column while the latent PPOs passed through the column and eluted immediately in the flowthrough. The protein concentrations were determined according to the Lambert-Beer law and their absorption at 280 nm using the extinction coefficient provided by ExPASy ProtParam.[13,14]

3. Heterologous Expression and Purification of recombinant PPO1 from *Malus domestica* (*Md*PPO1).

*Md*PPO1 was heterologously expressed in *E. coli* and purified by affinity chromatography following the protocol given in [15]. The *Md*PPO1 gene was N-terminally fused with the GST-tag of the pGEX-6P-1 vector. (GST-*Md*PPO1) was efficiently overexpressed using the synthetic tac promoter of the pGEX-6P-1 vector. *E. coli* was grown in 2xYT medium supplemented with ampicillin (100 µg/ml). *Md*PPO1 *E. coli* BL21 expression batches were inoculated with saturated overnight cultures and were grown at 37 °C under shaking for 4 hours until the OD_{600} reached a value between 0.6 and 0.8. Afterwards, the temperature was reduced to 20 °C and the cultures were induced with 0.5 mM isopropyl β-D-1-thiogalactopyranoside and 0.5 mM $CuSO_4$. The expression cultures remained at 20 °C under shaking for 24 to 40 hours. When the OD_{600} reached a value of 7 to 10 the cultures were collected by centrifugation at 10000 × g for 25 minutes at 4 °C. Lysis of the cells was performed by the freeze-thaw technique using liquid nitrogen. The lysates

were centrifuged at 10000 × g for 1 hour at 4 °C. The chromatographic purifications were carried out using an Äkta Purifier (GE Healthcare) placed in a refrigerator at 4 °C. The filtrated lysates were placed in a 50 ml injection loop and applied to a prepacked 5 ml GSTrap FF column. The target proteins were eluted with 50 mM Tris-HCl pH 7.5, 200 mM NaCl and 15 mM reduced glutathione. The GST-fusion protein fraction was concentrated using a Vivaspin® ultrafiltration device with a 30 kDa molecular weight cut-off (VWR). The samples were mixed with GST-HRV3C produced in-house at a mass ratio of 1 to 50 (protease to fusion protein) then again applied to a 5 ml GSTrap FF column, whereby the GST protein and the GST-tagged protease were still trapped by the column while the latent PPOs passed through the column and eluted.

4. Heterologous Expression, Purification and Activation of recombinant PPO4 from *Agaricus bisporus* (*Ab*PPO4).

Latent *Ab*PPO4 was expressed heterologously, purified by affinity chromatography and activated by limited proteolysis with Proteinase K (yielding *Ab*PPO4-act) as described in [12].

5. Enzyme Kinetics and Enzyme Activity Assays.

For the determination of the enzymatic activities of *Lt*PPO, *Md*PPO1 and *Ab*PPO4 and *Ab*PPO4-act, the appearance of the chromophore quinone products from (+) and (-)-larreatricin was detected spectrophotometrically. Absorption curves and spectra were recorded at 25 °C in a 96 well microplate on a TECAN infinite M200 (Tecan). (+) and (-)-larreatricin was insoluble in the aqueous buffer and in order to measure kinetics the substrates were solubilized by adding methanol. Kinetic measurements were done in a total volume of 200 µl, containing 50 mM Tris-HCl buffer pH 7.0, different volumes of methanol, different molarities of substrates, 5 mM 3-methyl-2-benzothiazolinone hydrazone (MBTH) in order to trap the formed quinones and different molarities of the enzymes and sodium dodecyl sulphate (SDS) for the latent enzymes in order to acquire full activity. For *Lt*PPO 1.5 mM SDS and 7 % methanol were used, for *Md*PPO1 3 mM SDS and 28 % methanol were used and kinetics for latent and active *Ab*PPO4 were determined in 2 mM SDS (latent enzyme only) and 28 % methanol (Figure S5).

Additionally, the molar absorption coefficient (ε at λ_{max}) of the chromophores formed via the enzymatic hydroxylation and oxidation of the (+) and (-)-larreatricin with *Ab*PPO4-act in 50 mM Tris buffer at pH 7.0 containing 5 mM MBTH was determined. The oxidation was carried out enzymatically with various amounts of substrate (in the µM range), oxidised by *Ab*PPO4-act (also in the µM range) and the formation of the MBTH-quinone adducts was monitored spectrophotometrically. The molar absorption coefficient was determined by linear regression at the appropriate wavelength (λ_{max}) (Figure S6). Spectra were taken routinely for each substrate on a Shimadzu UV-1800 spectrophotometer (Shimadzu Deutschland, Duisburg, Germany) in 1 ml of solution at 25 °C.

6. Tables and Figures

Table S8. **Primers used to amplify the gene for larreatricin hydroxylase**

Primers	Accession number	Size [bp]	ORF [AA]*	MW [kDa]
**fw:** 5´ AgcggcTcTTcAATgGCTCCAATTCAACCACCG 3´<br>**rev:** 5´AgcggcTcTTcTcccATCTTCCTCATCTCTAACAAATTCAATC 3´	AY370019	1515	505	57.7

* number of translated amino acids in the open reading frame

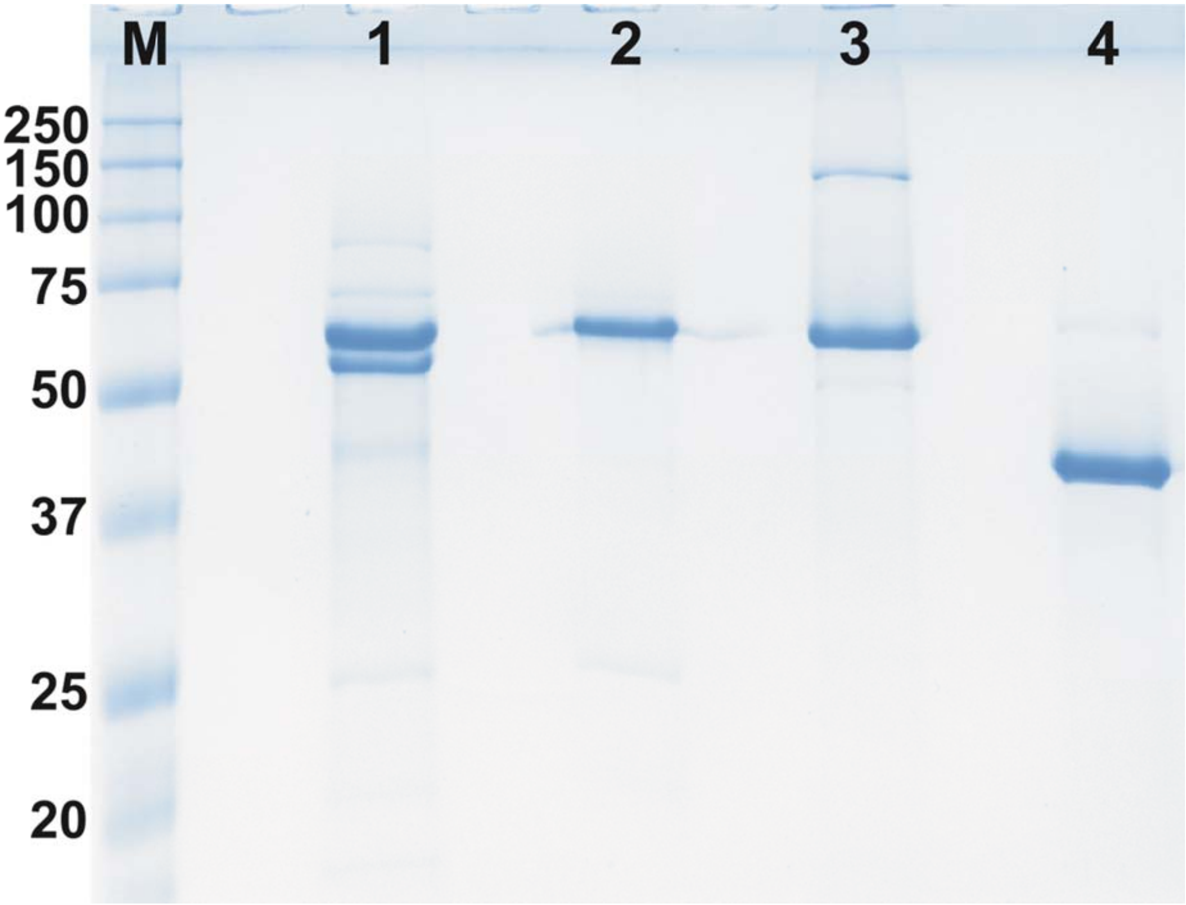

Figure S4. SDS-PAGE gel of pure enzymes. 1) 5 µg of larreatricin hydroxylase. 2) 5 µg of *Md*PPO1. 3) 5 µg of latent *Ab*PPO4 . 4) 5 µg of activated *Ab*PPO4. M) Molecular weight marker (in kDa)

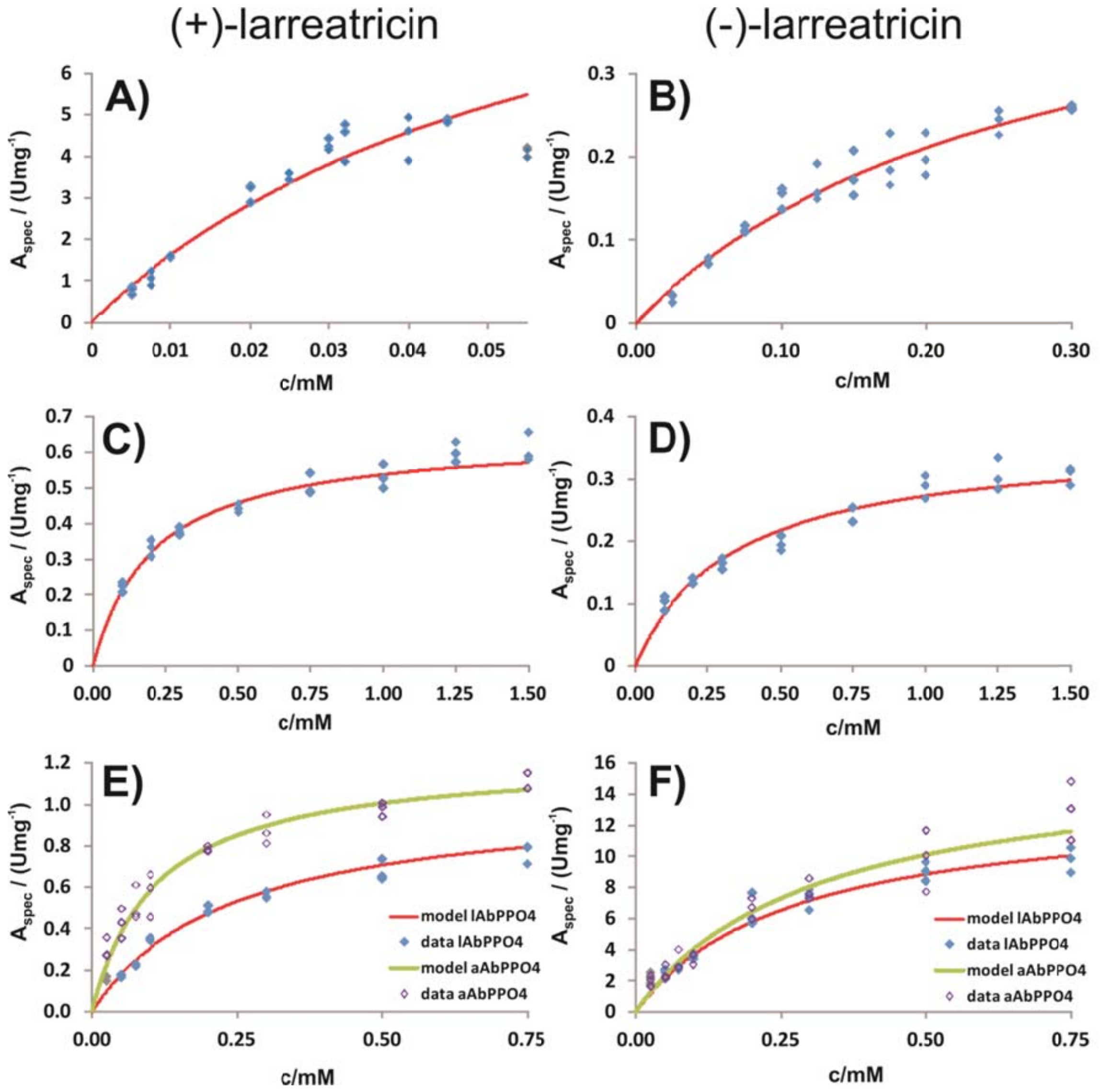

Figure S5. Michaelis-Menten diagrams for the substrates in Table 1. A) *Lt*PPO on (+)-larreatricin, B) *Lt*PPO on (-)-larreatricin, C) *Md*PPO1 on (+)-larreatricin, D) *Md*PPO1 on (-)-larreatricin, E) latent (l*Ab*PPO4) and active (a*Ab*PPO4) *Ab*PPO4 on (+) larreatricin, F) latent (l*Ab*PPO4) and active (a*Ab*PPO4) *Ab*PPO4 on (-)-larreatricin. Blue, purple and grey diamonds represent the measured slopes, grey data points have been excluded from the data analysis yielding the least-squares optimized parameters of the Michaelis-Menten models. Reaction rates predicted using those models are shown as red (based on latent enzyme forms) and green (based on active enzyme form) curves.

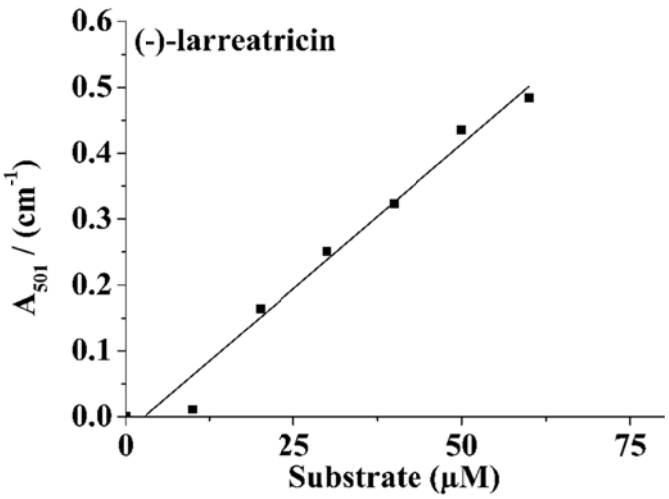

Figure S6. Absorbances at 501 nm obtained by (hydroxylation and) oxidation of the phenolic substrate (-)-larreatricin in the presence of MBTH.

References

[1] D. Marcoux, P. Bindschädler, A. W. H. Speed, A. Chiu, J. E. Pero, G. A. Borg, D. A. Evans, *Org. Lett.* **2011**, *13*, 3758-3761.

[2] J. K. Crandall, T. Schuster, *J. Org. Chem.* **1990**, *55*, 1973-1975.

[3] P. Magnus, N. Sane, B. P. Fauber, V. Lynch, *J. Am. Chem. Soc.*, **2009**, *131*, 16045-16047.

[4] S. G. A. Moinuddin, S. Hishiyama, M.-H. Cho, L. B. Davin, N. G. Lewis, *Org. Biomol. Chem.* **2003**, *1*, 2307-2313.

[5] J. B. Johnson, E. A. Bercot, C. M. Williams, T. Rovis, *Angew. Chem. Int. Ed.* **2007**, *46*, 4514-4518.

[6] Bruker SAINT v7.68A & 8.32B, Copyright © 2005-**2018** Bruker AXS.

[7] Sheldrick G.M. (1996). SADABS. University of Göttingen, Germany.

[8] Sheldrick G.M. (2015). SHELXS,SHELXL. University of Göttingen, Germany.

[9] O.V. Dolomanov, L. J. Bourhis, R. J. Gildea, J. A. K. Howard, H. Puschmann, *J. Appl. Cryst.* **2009**, *42*, 339-341.

[10] C.B. Huebschle, G. M. Sheldrick, B. Dittrich, *J. Appl. Cryst.* **2011**, *44*, 1281-1284.

[11] K. Brandenburg, DIAMOND, Version 3.2c, *Crystal Impact GbR*, Bonn, Germany, **2009**.

[12] M. Pretzler, A. Bijelic, A. Rompel, *Sci. Rep.* **2017**, 7, 1810.

[13] E. Gasteiger, C. Hoogland, A. Gattiker, S. Duvaud, M. Wilkins, R. Appel, A. Bairoch, in Proteomics Protoc. Handb. (Ed.: J. Walker), *Humana Press*, **2005**, pp. 571-607.

[14] D. F. Swinehart, *J. Chem. Educ*. **1962**, 39, 333.

[15] I. Kampatsikas, A. Bijelic, M. Pretzler, A. Rompel, *Sci. Rep.* **2017**, 7, 8860.
